# Rigidity‐Driven Structural Isomers in the NaCl–Ga_2_S_3_ System: Implications for Energy Storage

**DOI:** 10.1002/smsc.202400371

**Published:** 2024-10-01

**Authors:** Maria Bokova, Mohammad Kassem, Takeshi Usuki, Andrey Tverjanovich, Anton Sokolov, Daniele Fontanari, Alex C. Hannon, Chris J. Benmore, Igor Alekseev, Shinji Kohara, Pascal Roussel, Maxim Khomenko, Koji Ohara, Yohei Onodera, Arnaud Cuisset, Eugene Bychkov

**Affiliations:** ^1^ Laboratoire de Physico‐Chimie de l’Atmosphère Université du Littoral Côte d’Opale Dunkerque 59140 France; ^2^ Faculty of Science Yamagata University Yamagata 990‐8560 Japan; ^3^ Institute of Chemistry St. Petersburg State University St. Petersburg 198504 Russia; ^4^ ISIS Facility Rutherford Appleton Laboratory Chilton Didcot OX11 0QX UK; ^5^ X‐ray Science Division Advanced Photon Source Argonne National Laboratory Lemont IL 60439 USA; ^6^ Quantum Beam Diffraction Group Center for Basic Research on Materials National Institute for Materials Science 1‐1‐1 Kouto, Sayo‐cho, Sayo‐gun Hyogo 679‐5148 Japan; ^7^ Unité de Catalyse et de Chimie du Solide (UCCS) Université de Lille CNRS, Centrale Lille, Université d’Artois 59000 Lille France; ^8^ Faculty of Physics Lomonosov Moscow State University Moscow 119991 Russia; ^9^ Faculty of Materials for Energy Shimane University 1060, Nishi‐Kawatsu‐Cho Matsue Shimane 690‐8504 Japan

**Keywords:** fast sodium halide conductors, network rigidity, sodium diffusion, structural isomers

## Abstract

Alternative energy sources require the search for innovative materials with promising functionalities. Systems with unusual chemical properties represent an insufficiently explored domain, concealing unexpected features. Using diffraction and Raman spectroscopy over a wide temperature range, supported by first‐principles simulations, a rare phenomenon is unveiled: phase‐dependent chemical interactions between binary components in the NaCl–Ga_2_S_3_ system. In this unique occurrence, previously intact binary crystalline species transform upon melting into mixed liquid structural isomers, forming bonds with new partners. The chemical combinatorics appears to be fully reversible for stable crystals and liquids. Despite this, rapidly frozen glasses out of thermodynamic equilibrium remain in a metastable isomeric state, offering remarkable properties, particularly a high room‐temperature Na^+^ conductivity, comparable to the best sodium halide superionic conductors and therefore encouraging for sodium solid‐state batteries and energy applications. A rigidity paradigm is responsible for the observed phenomenon, as the extremely constrained Ga_2_S_3_ crystal lattice does not survive viscous flow, breaking up at a short‐range level. The removal of rigidity constraints and dense packing leads to a significant increase in empty space, which is the origin of high sodium diffusivity. Broadly, the rigidity‐driven structural isomerism opens up an inspiring path to the discovery of atypical materials.

## Graphical abstract

A rare phenomenon—phase‐dependent chemical interactions—is observed in the eutectic NaCl–Ga_2_S_3_ system. Previously distinct binary crystalline species, NaCl and Ga_2_S_3_, transform upon melting into mixed structural isomers, forming bonds with new partners. This process is fully reversible, but supercooled liquids and glasses remain in a metastable isomeric form, offering high Na^+^ conductivity.

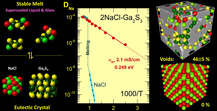

## 1 Introduction

Carbon neutral economy and reduction of fossil fuels for transportation require research and development in the fields of alternative energy sources and related materials. Fast alkali ion conductors for efficient high‐density solid‐state batteries represent a viable and cost‐effective path of reaching these goals.^[^ ^1^, ^2^, ^3^ ^]^ Lithium and sodium halide superionic conductors have become promising electrolytes for all‐solid‐state batteries.^[^ ^4^, ^5^ ^]^ They exhibit similar high‐voltage stability, air and moisture resistance in comparison with oxide counterparts but reveal much better ionic conductivity and mechanical deformability. The best sulfide solid electrolytes show record‐holding ion transport properties and excellent cell processability; however, they suffer from insufficient chemical stability and low electrochemical window, resulting in limited performance of the solid‐state cells.

Typical alkali halide ion conductors, such as ${\text{Li}}_{3}{\text{InCl}}_{6}$ or ${\text{Na}}_{3-x}Y_{1-x}{\text{Zr}}_{x}{\text{Cl}}_{6}$, are based on close‐packed or dense structures. Consequently, small Li^+^ cations exhibit high diffusion coefficients and room‐temperature ionic conductivity ($\sigma_{\text{RT}}$ ≈ 1 mS cm^−1^), while larger Na^+^ species encounter steric hindrances and show lower ionic mobility^[^ ^5^, ^6^, ^7^, ^8^ ^]^ resulting in $\sigma_{\text{RT}}$ ≲ 0.01 mS cm^−1^. Aliovalent substitutions, optimal fraction and size of vacancies, and a decrease in crystallinity were found to be favorable factors, increasing sodium ion transport. Recently, heterogeneous sodium solid electrolytes were reported,^[^ ^9^, ^10^ ^]^ combining dense high‐coordination and amorphous low‐coordination halide frameworks, reaching $\sigma_{\text{RT}}$ = 2.7 mS cm^−1^ with an activation energy of 0.26 eV. Nevertheless, the search for novel sodium halide superionic conductors is far from over and requires systematic studies of new systems with unusual chemical phenomena.

The common level of Na^+^ conductivity, $\sigma_{\text{RT}}$ ≈ 0.003 mS cm^−1^, can also be achieved in the NaCl–Ga_2_S_3_–GeS_2_ glasses with quite low sodium chloride content of about 20 mol% (6.4 at% Na).^[^ ^11^ ^]^ Similar alkali halide systems are promising for optical applications^[^ ^12^, ^13^, ^14^ ^]^ and exhibit unusual structural properties: a mixed gallium and alkali environment in the vitreous state^[^ ^15^, ^16^, ^17^ ^]^ in contrast to the pure sulfide or halide local structure in their crystalline counterparts. This mixed environment appears to be beneficial for the functional properties of these materials and also raises a fundamental question related to a possible difference in chemical interactions between thermodynamically stable crystalline solids and liquids, considering that glass is a frozen supercooled liquid out of thermodynamic equilibrium.

The system of interest is NaCl–Ga_2_S_3_, which exhibits a phase diagram of eutectic type,^[^ ^18^ ^]^ implying no chemical interactions between NaCl and Ga_2_S_3_, at least in the crystalline state. However, using neutron and synchrotron diffraction, accompanied by Raman spectroscopy measurements and first‐principles simulations, we will show that the 2NaCl–Ga_2_S_3_ liquid behaves differently. The binary components (sodium chloride and gallium sesquisulfide), which remain chemically intact in the crystalline form, interact in the stable liquid, forming structural isomers, where the same atomic species have different partners. Surprisingly, the process is fully reversible, and the binary components appear to be restored, crystallizing upon either slow cooling or fast quenching. Nevertheless, the simulations reveal that the frozen supercooled liquid or glass remains in a metastable isomeric form and shows promising functional properties, in particular, high ionic conductivity and sodium diffusion, comparable to the best sodium halide superionic conductors. The excessive rigidity of crystalline gallium (III) sulfide seems to be a driving force behind its atypical chemical behavior.

## 2 Results and Discussion

### 2.1 Absence of Chemical Interactions Between Binary Components in Crystalline NaCl–Ga_2_S_3_

**Figure 1:**
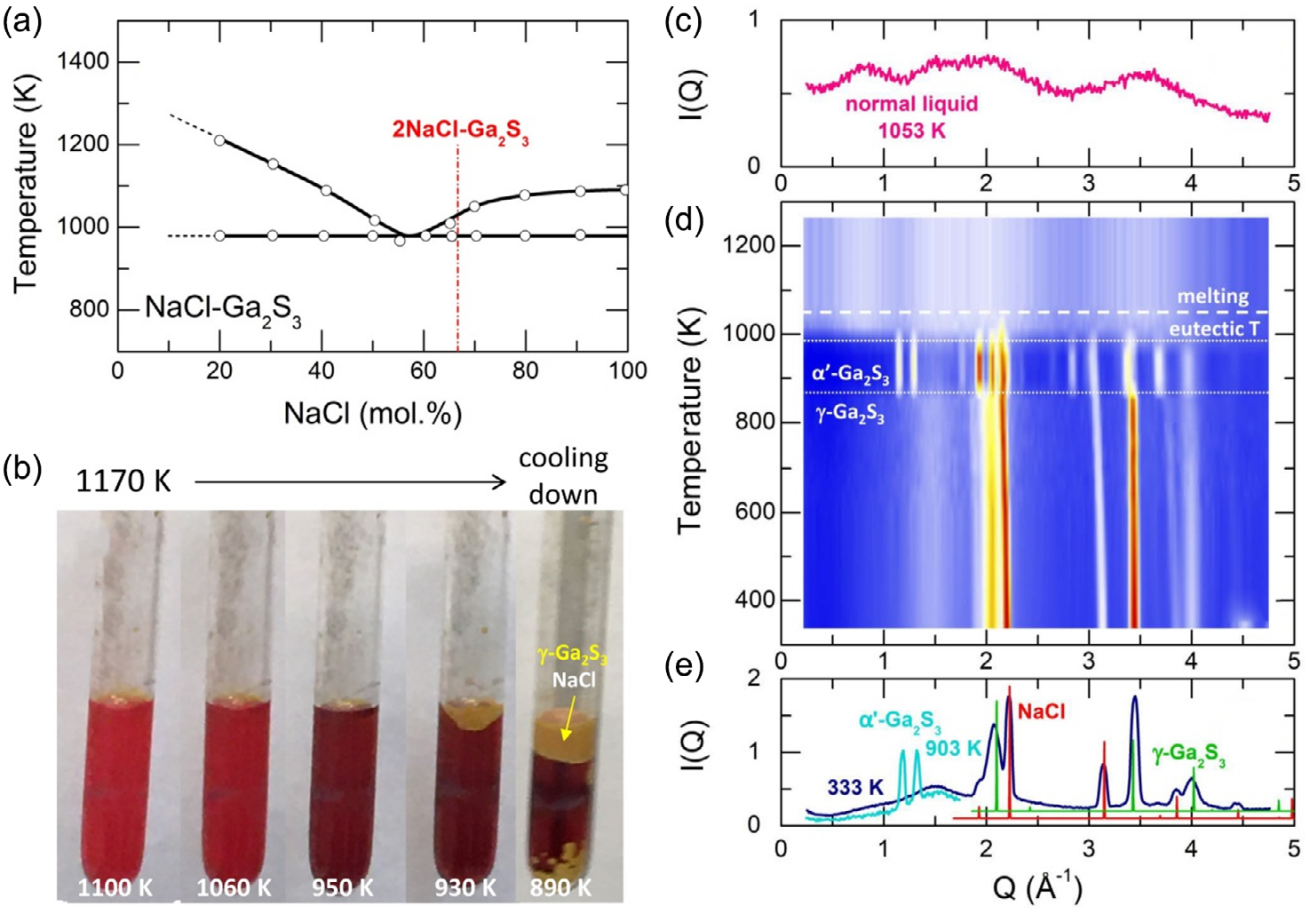
a) Phase diagram of NaCl–Ga_2_S_3_; b) fast cooling of the stable 2NaCl–Ga_2_S_3_ melt yields a polycrystalline mixture of NaCl/γ‐Ga_2_S_3_; HE‐XRD measurements of 2NaCl–Ga_2_S_3_ over a wide temperature range: c) normal liquid at 1053 K; d) color‐coded 2D diffraction patterns between 333 and 1223 K; e) crystalline samples at 333 and 903 K (only a low‐*Q* part at *Q* ≤ 1.7 Å^−1^ is shown at 903 K to reveal the characteristic ($110/1\bar{1}0$) and ($200/\bar{1}11$) Bragg peaks of α′‐Ga_2_S_3_). The Bragg peaks of NaCl and γ‐Ga_2_S_3_ references are also displayed in (e). See also Figure S1, Supporting Information.

As mentioned above, the phase diagram NaCl–Ga_2_S_3_ appears to be a conventional eutectic‐type phase diagram (**Figure** **1**a),^[^ ^18^ ^]^ indicating no chemical interaction between the binary components. In situ high‐energy X‐ray diffraction (HE‐XRD) measurements as a function of temperature support this finding, revealing only the Bragg peaks of phase‐separated cubic NaCl (space group $Fm\bar{3}m$)^[^ ^19^ ^]^ and γ‐Ga_2_S_3_ ($F\bar{4}3m$)^[^ ^20^ ^]^ (Figure 1d,e). Above 853 K, cubic γ‐Ga_2_S_3_ transforms into the monoclinic polymorph α’‐Ga_2_S_3_ (space group Cc)^[^ ^21^ ^]^ before melting at the eutectic temperature. The temperature of the γ−α’ transition is consistent with previous reports for pure gallium(III) sulfide.^[^ ^20^, ^22^ ^]^

Fast quenching of the stable 2NaCl–Ga_2_S_3_ melt (Figure 1c) in icy water yields a polycrystalline mixture of NaCl/γ‐Ga_2_S_3_ (Figure 1b). Slow cooling produces NaCl and α′‐Ga_2_S_3_ (see the structure factors $S_{X}(Q)$ and total correlation functions $T_{X}(r)$ in Figure S2 and S3, Supporting information). Similar absence of chemical interactions was previously observed in the PbCl_2_–Ga_2_S_3_ system (the phase diagram of eutectic type)^[^ ^22^ ^]^ and KI‐Ga_2_S_3_ alloys.^[^ ^23^ ^]^

**Figure 2:**
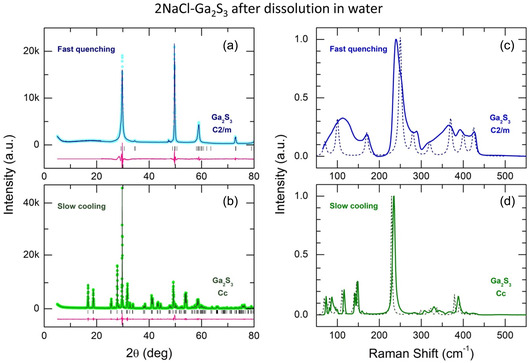
LeBail refinement and Raman spectra of a,c) fast quenched and b,d) slowly cooled 2NaCl–Ga_2_S_3_ melt following the sodium chloride dissolution in water. The dashed lines in (c) and (d) represent Raman spectrum of γ‐Ga_2_S_3_, obtained by decompression from 23.7 GPa,^[^ ^40^ ^]^ and density functional theory (DFT) ‐ derived spectrum of α′‐Ga_2_S_3_.^[^ ^41^ ^]^ See the text for further details.

Sodium chloride dissolution in water allows for the precise crystallographic determination of the Ga_2_S_3_ lattice constants in these two cases. As expected, the slow cooling results in the monoclinic α’‐Ga_2_S_3_ polymorph (*a* = 11.1269(7) Å, *b* = 6.4121(4) Å, *c* = 7.0378(4) Å, *α* = 90°, *β* = 121.189(3)°, *γ* = 90°), **Figure** **2**b and S4, Supporting Information, in good agreement with the reference compound.^[^ ^21^ ^]^ In contrast, the fast quenched 2NaCl–Ga_2_S_3_ melt generates a slightly different polymorph than the expected cubic γ‐Ga_2_S_3_.^[^ ^20^ ^]^ Basically, the diffraction pattern cannot be fitted by a single cubic phase (Figure S5, Supporting Information), and better agreement is provided by the monoclinic lattice (space group C2/m): *a* = 6.218(5) Å, *b* = 3.6619(9) Å, *c* = 3.6552(10) Å, *α* = 90°, *β* = 123.50(7)°, *γ* = 90° (see Figure 2a and S6, Supporting Information). The hypothesis of two cubic phases with similar lattice constants is also possible (Figure S7, Supporting Information); however, the fit quality is slightly worse. Further details on the LeBail analysis can be found in the Supporting Information. In the following, we will refer to this monoclinic polymorph as *γ*′‐Ga_2_S_3_.

A drastic difference between the two polymorphs was also found in the Raman spectra (Figure 2c,d); the most visible changes are related to a different width of the vibration modes.

### 2.2 Neutron and HE‐XRD of Liquid 2NaCl–Ga_2_S_3_

**Figure 3:**
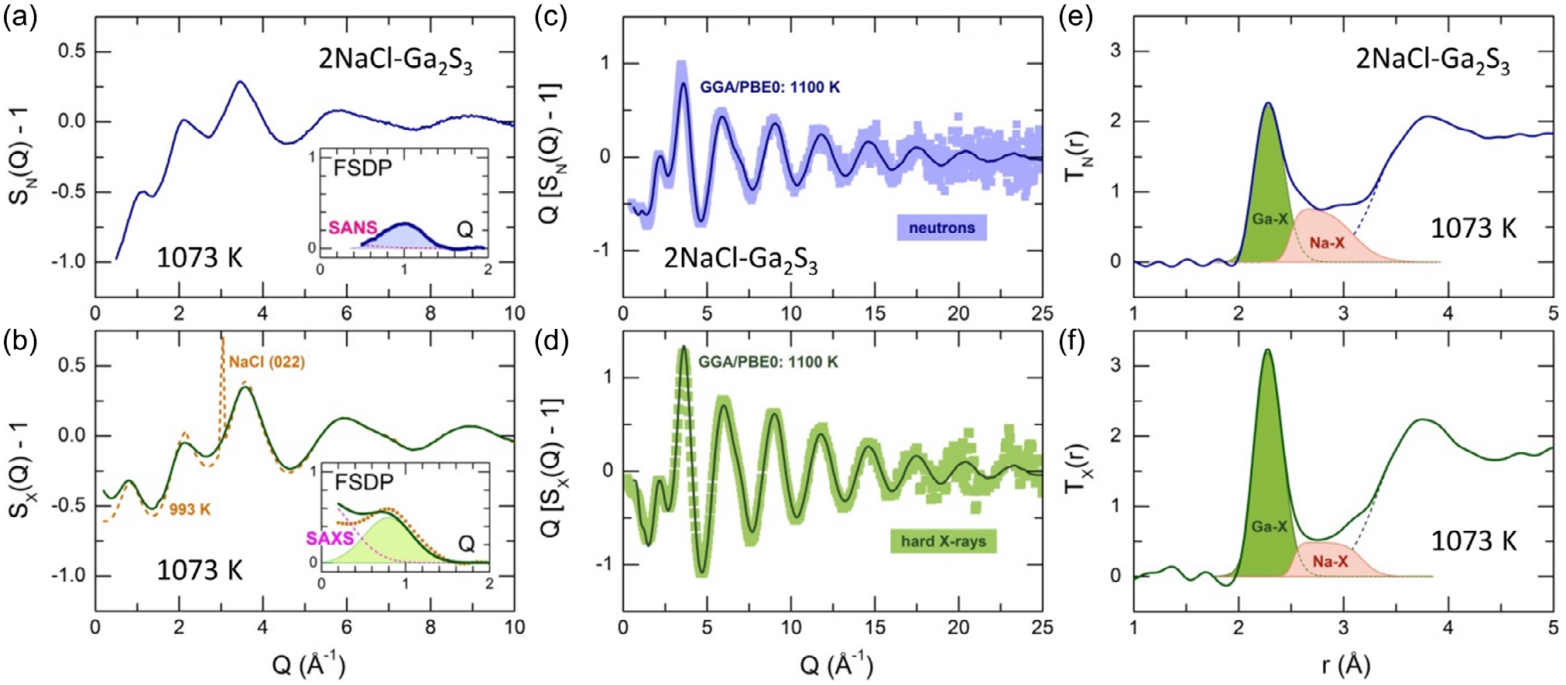
Diffraction data of liquid 2NaCl–Ga_2_S_3_: a) experimental neutron $S_{N}\left(Q\right)$ and b) X‐ray $S_{X}\left(Q\right)$ structure factors at 1073 K; the insets show isolated FSDPs (highlighted in light blue and green) and small‐angle scattering after background subtraction;^[^ ^76^, ^77^ ^]^ $S_{X}\left(Q\right)$ of the eutectic liquid with remaining cubic NaCl traces at 993 K is also shown in (b) by the dashed cinnamon line; experimental and FPMD‐derived c) neutron $Q[S_{N}\left(Q\right)-1]$ and d) X‐ray $Q[S_{X}\left(Q\right)-1]$ interference functions; the color‐coded squares represent ND and HE‐XRD data and the solid lines represent the FPMD results; fitting e) neutron $T_{N}\left(r\right)$ and f) X‐ray $T_{X}\left(r\right)$ total correlation functions using asymmetric functions for unresolved Ga–X (highlighted in green) and Na–X (light red) contributions (X = S and Cl).

Even partial melting above the eutectic temperature changes the local environment of gallium and sodium in 2NaCl–Ga_2_S_3_ (Figure S8, Supporting Information). The liquid‐like X‐ray structure factor $S_{X}\left(Q\right)$ at 993 K still exhibits the (022) Bragg peak of sodium chloride and remnants of the (002), (024), and (224) reflexes (**Figure** **3**b), but the total correlation function $T_{X}\left(r\right)$ appears to be drastically different compared to the crystalline counterpart at 913 K (Figure S8, Supporting Information). Considering the similar atomic sizes of sulfur and chlorine, a complementary use of neutrons and hard X‐rays was necessary for diffraction studies. Neutrons are more sensitive to chlorine, ${\bar{b}}_{\text{Cl}}/{\bar{b}}_{\text{Ga}}$ = 1.314, while X‐rays to gallium, $Z_{\text{Cl}}/Z_{\text{Ga}}$ = 0.548, where ${\bar{b}}_{i}$ is the neutron coherent scattering length and $Z_{i}$ is the atomic number.

Typical neutron $S_{N}\left(Q\right)$ and X‐ray $S_{X}(Q)$ structure factors of the stable liquid 2NaCl–Ga_2_S_3_ at 1073 K are shown in Figure 3. An overall similarity in the shape of $S_{N}\left(Q\right)$ and $S_{X}(Q)$ is noted, with characteristic differences in the amplitude and position of the first sharp diffraction peak (FSDP) at 0.8 ≤ $Q_{0}$ ≤ 1.0 Å^−1^. The higher FSDP intensity in $S_{X}(Q)$ seems to be related to a greater involvement of the Ga‐related partial structure factors $S_{\text{GaX}}\left(Q\right)$ in the FSDP, as their *Q*‐dependent X‐Ray weighting coefficients are larger than the neutron counterparts, $w_{\text{GaX}}^{X}\left(Q\right)$ > $w_{\text{GaX}}^{N}$, where X = Ga, S, and Na. The only exception is $w_{\text{GaCl}}^{X}\left(Q\right)$. Multiple contributions to the FSDP with contrasting weightings explain the difference in position for neutrons ($Q_{0}^{N}$ = 1.0 Å^−1^) and X‐rays ($Q_{0}^{X}$ = 0.8 Å^−1^) at 1073 K.

Additionally, temperature‐dependent small‐angle X‐Ray scattering (SAXS) at *Q* < 0.8 Å^−1^ is observed, while small‐angle neutron scattering is hardly visible at low *Q*, partly due to the different $Q_{\text{min}}$ values: 0.2 Å^−1^ for HE‐XRD and 0.5 Å^−1^ for neutron diffraction (ND). Mesoscopic inhomogeneity associated with Ga could explain the SAXS contribution.

The correlation functions in *r*‐space were obtained using the usual Fourier transform

(1) $$T_{N/X}\left(r\right)=4\pi \rho_{0}r+\frac{2}{\pi }\int_{0}^{Q_{\text{max}}}Q\left[S_{N/X}\left(Q\right)-1\right]\sin\left(Qr\right)M\left(Q\right)dQ$$

where $T_{N/X}\left(r\right)$ is the neutron or X‐ray total correlation function, $\rho_{0}$ is the number density, and M(Q) is the Lorch modification function. In contrast to polycrystalline mixtures NaCl/α′‐Ga_2_S_3_ and NaCl/γ‐Ga_2_S_3_, which exhibit well‐separated Ga–S and Na–Cl nearest‐neighbor (NN) peaks at 2.24–2.28 and 2.9 Å, respectively, and the coordination numbers $N_{\text{GaS}}$ = 4 and $N_{\text{NaCl}}$ = 6 (Figure S2 and S3, Supporting Information), both $T_{N}\left(r\right)$ and $T_{X}\left(r\right)$ of liquid 2NaCl–Ga_2_S_3_ are different (Figure 3e,f). As expected, the NN peaks are broad and unresolved. However, a simple 3‐peak Gaussian fitting of $T_{N}\left(r\right)$, assuming Ga–S NNs at 2.28 Å, Na–Cl NNs at 2.79 Å, and unresolved second neighbors at 3.7 Å, yields unrealistic and astonished values of $N_{\text{GaS}}$ = 5.3 and $N_{\text{NaCl}}$ = 1.2. In other words, the model suggesting the absence of chemical interactions between Ga_2_S_3_ and NaCl in the liquid phase, following the phase diagram, is incorrect. The obtained values of coordination numbers and a strong N/X contrast for the constituent species assume the exchange reactions, which can be rationalized as follows: Na–Cl + Ga–S ⇄ Na–S + Ga–Cl. Consequently, the NN peak at 2.28 Å would contain Ga–S and Ga–Cl contributions, and the NN peak at 2.79 Å consists of Na–Cl and Na–S correlations.

The appropriate fitting of both $T_{N}\left(r\right)$ and $T_{X}\left(r\right)$ functions faces two major difficulties: 1) the asymmetric shape of the NN peaks, partly related to similar but not identical contributions of Ga–S (2.20–2.35 Å)^[^ ^17^, ^21^, ^24^, ^25^ ^]^ and Ga–Cl (2.10–2.34 Å),^[^ ^26^, ^27^ ^]^ as well as Na–Cl (2.66–2.90 Å)^[^ ^19^, ^28^ ^]^ and Na–S (2.77–2.90 Å)^[^ ^29^, ^30^, ^31^ ^]^ correlations, and 2) the unknown ratio of Ga–S/Ga–Cl and Na–Cl/Na–S populations. As a first step, fitting with symmetric functions was used, varying the Cl/S proportion for both gallium and sodium local environment, which was set to be identical for neutron and X‐rays. Then, the best values of Ga–S/Ga–Cl and Na–Cl/Na–S ratios were constrained, allowing fitting with asymmetric functions. The derived results are shown in Figure 3e,f and collected in **Table** **1**. Gallium and sodium were found to be fourfold coordinated with different fractions of the Cl/S mixed neighbors. Gallium predominantly possesses a sulfide environment (82%), while sodium has slightly more Cl NNs (53%).

**Table 1:** Interatomic distances $r_{ij}$ (Å) and coordination numbers $N_{ij}$ in liquid 2NaCl–Ga_2_S_3_ derived from the least‐square fitting of the experimental datasets and first‐principles molecular dynamics.

S−S		Ga−S		Ga−Cl		Ga−Ga		$N_{\text{Ga}-X}$	Na−Cl		Na−S		$N_{\text{Na}-X}$
$r_{ij}$	$N_{ij}$	$r_{ij}$	$N_{ij}$	$r_{ij}$	$N_{ij}$	$r_{ij}$	$N_{ij}$		$r_{ij}$	$N_{ij}$	$r_{ij}$	$N_{ij}$	
ND													
−	−	2.28a)	3.29	2.28a)	0.70	−	−	3.99	2.73b)	2.1	2.73b)	1.9	4.0
HE‐XRD													
−	−	2.28a)	3.22	2.28a)	0.68	−	−	3.90	2.77b)	2.0	2.77b)	1.8	3.8
FPMD													
2.08	0.08	2.24c)	3.23c)	2.23c)	0.60c)	2.40	0.09	3.92	2.67d)	2.6d)	2.83d)	1.8d)	4.4d)

a) Unresolved contributions of the Ga−S and Ga−Cl NN correlations;

b) Unresolved contributions of strongly asymmetric Na−Cl and Na−S NN contacts;

c) Slightly asymmetric peaks;

d) Strongly asymmetric peaks; the uncertainties in Ga–X and Na–X distances are ±0.02 and ±0.05 Å, respectively; the uncertainties in gallium and sodium local coordination are ±0.05 and ±0.20.

We should note a different shape of asymmetric Na–X NN correlations for neutrons and hard X‐rays. A more intense unresolved contribution at lower *r* in the $T_{N}\left(r\right)$ function suggests that the maximum of the Ga–Cl NN contacts is slightly shifted to lower distances compared to that of the Na–S counterparts. This difference is associated with a dissimilar sensitivity of neutrons and X‐rays: ${\bar{b}}_{\text{Cl}}/{\bar{b}}_{S}$ = 3.364 and $Z_{\text{Cl}}/Z_{S}$ = 1.0625.

### 2.3 FPMD of Liquid 2NaCl–Ga_2_S_3_

**Figure 4:**
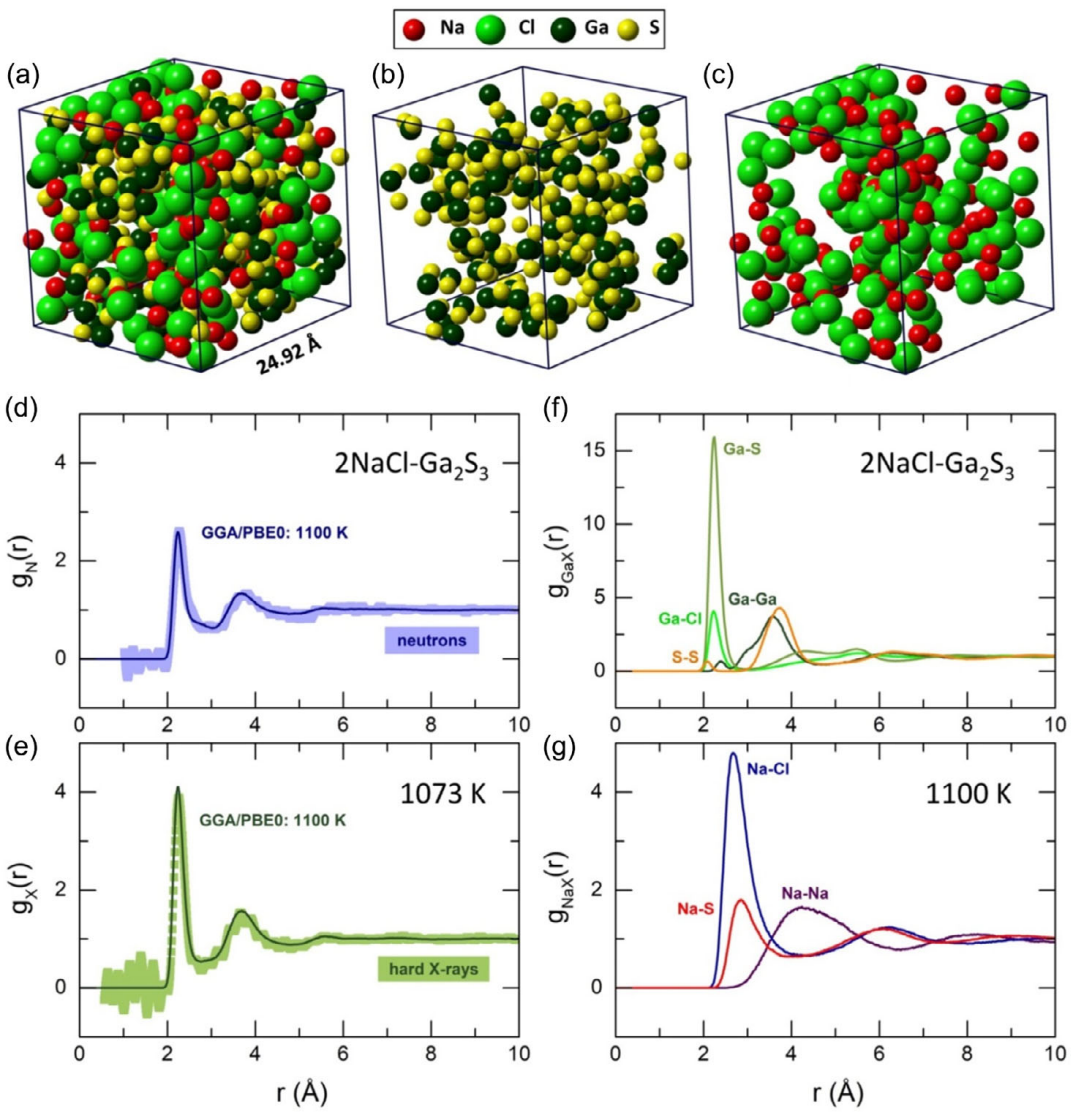
FPMD simulation box containing 495 atoms (110 Na, 110 Cl, 110 Ga, and 165 S): a) full box, b) Ga and S species, c) Na and Cl distributions; experimental and FPMD‐derived d) neutron $g_{N}\left(r\right)$ and e) X‐ray $g_{X}\left(r\right)$ pair‐distribution functions; the color‐coded squares represent experimental data and the solid lines denote the FPMD results; partial pair‐distribution functions for f) Ga–S, Ga–Cl, Ga–Ga, and S–S atomic pairs, and g) Na–Cl, Na–S, and Na–Na correlations for stable liquid 2NaCl–Ga_2_S_3_.

The applied Born–Oppenheimer molecular dynamics in the generalized gradient approximation,^[^ ^32^ ^]^ using the hybrid exchange‐correlation functional PBE0^[^ ^33^, ^34^ ^]^ (GGA–PBE0), appears to be efficient and precise for liquid 2NaCl**–**Ga_2_S_3_, as for other chalcogenide and chalcohalide systems.^[^ ^35^, ^36^, ^37^, ^38^ ^]^ The first‐principles molecular dynamics (FPMD)‐derived neutron and X‐ray interference functions $Q[S_{N/X}\left(Q\right)-1]$, shown in Figure 3c,d, reveal good agreement with the experimental data both in amplitude and position of the diffraction features. We should also note an enhanced SAXS signal in computed data, although the size of the simulation box (24.92 × 24.92 × 24.92 Å^3^), containing 495 atoms, seems to be insufficient to capture mesoscopic inhomogeneities. An approximate nonuniform distribution of the components can be observed in snapshots of the simulation box (**Figure** **4**a–c).

As expected, the FPMD real‐space functions also closely resemble the ND and HE‐XRD experimental results (Figure 4d,e). The derived Ga–X and Na–X partial pair‐distribution functions $g_{ij}\left(r\right)$ (Figure 4f,g) are consistent with the previous analysis, indicating a mixed gallium and sodium environment in liquid 2NaCl–Ga_2_S_3_ (Table 1). Additionally, the NN peak at 2.28 Å contains a small fraction of S–S ($N_{\text{SS}}$ = 0.08) and Ga–Ga ($N_{\text{GaGa}}$ = 0.09) homopolar bonds. These contributions at 2.08 and 2.40 Å, respectively, are not visible in experimental data due to their weak amplitude.

**Figure 5:**
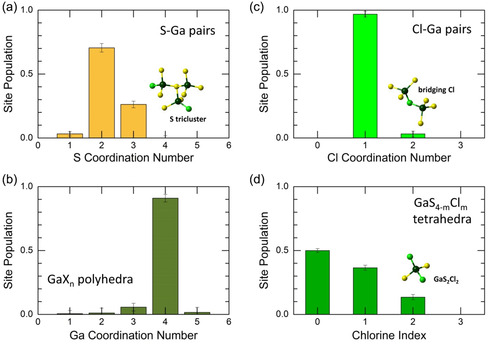
FPMD‐derived local coordination in liquid 2NaCl**–**Ga_2_S_3_ at 1100 K: coordination number distributions of a) sulfur $N_{\text{SGa}}$, b) gallium $N_{\text{GaX}}$ (X = S, Cl, Ga), and c) chlorine $N_{\text{ClGa}}$; d) chlorine index: number of Cl species in mixed ${\text{GaS}}_{4-m}S_{m}$ tetrahedra. The insets show sulfur triclusters, bridging chlorine, and mixed tetrahedron GaS_2_Cl_2_.

Local coordination of gallium, sulfur, and chlorine is shown in **Figure** **5**. The average gallium coordination, $N_{\text{GaX}}$ = 3.92 ± 0.05 (Table 1), indicates a predominant contribution of fourfold coordinated Ga_4F_ species (91 ± 3%). The fraction of trigonal gallium Ga_3F_ is significantly lower (6 ± 3%), while the population of other under‐ and overcoordinated Ga atoms is statistically irrelevant.

The twofold sulfur coordination S_2F_ is clearly dominant (70 ± 3%). Sulfur triclusters, that is, trigonal sulfur S_3F_ connected to three Ga central atoms (see the inset in Figure 5a), constitute the second most populated species (26 ± 3%). The fraction of terminal S(*t*) hardly exceeds the statistical limit. In contrast, terminal chlorine Cl(*t*) is overwhelmingly populated (97 ± 2%), while twofold coordinated bridging Cl(*b*) species (the inset in Figure 5c) appear to be negligibly small. One half of GaX_4_ tetrahedra lack chlorine (Figure 5d). The remaining part is subdivided into two groups: the population of GaS_3_Cl units (36 ± 2%) is markedly higher than that of GaS_2_Cl_2_ entities (14 ± 2%).

As previously reported for sodium sulfide and sodium chloride systems,^[^ ^28^, ^30^, ^31^ ^]^ all real‐space partial functions $g_{\text{NaX}}\left(r\right)$ are strongly asymmetric. The prominent asymmetry observed in both experimental (Figure 3e,f) and FPMD‐derived Na–Cl and Na–S correlations (Figure 4g) complicates the precise determination of sodium local coordination. Several approaches were used. 1) Taking Na–Cl and Na–S crystalline cutoffs in cubic sodium chloride and sulfide (3.35 Å), the average sodium coordination appears to be $N_{\text{NaX}}$ = 3.7. 2) Extending the cutoff to 3.56 Å, representing the border value between nearest (Na–Cl, Na–S) and second (Na–Na, Na–Ga) neighbors, yields $N_{\text{NaX}}$ = 4.2. 3) Finally, the observed minimum in real‐space partials $g_{\text{NaX}}\left(r\right)$ or $T_{\text{NaX}}\left(r\right)$ at 4.0 Å suggests $N_{\text{NaX}}$ = 5.2. Considering the above estimations, the best assessment of the sodium local coordination appears to be $N_{\text{NaX}}$ = $N_{\text{NaCl}}$ + $N_{\text{NaS}}$ = 2.6 + 1.8 = 4.4 ± 0.3. This value is consistent with sodium coordination in liquid NaCl, 4.7 ± 0.1,^[^ ^28^ ^]^ and Na_2_S, 4.0 ± 0.2,^[^ ^31^ ^]^ and slightly larger than the experimental fitting results (Table 1). Additionally, there is a difference in the higher proportion of the FPMD‐derived Na–Cl environment (59%) versus the experimental data analysis (53%).

The different shape of experimental unresolved Na–X NN correlations in $T_{N}\left(r\right)$ and $T_{X}\left(r\right)$ datasets (Figure 3), attributed to a shift of the Na–Cl maximum to lower *r*, is confirmed by FPMD. The Na–Cl and Na–S partials are peaked at 2.67 and 2.83 Å, respectively (Table 1). Additionally, the Na–Cl value is consistent with that in molten sodium chloride.^[^ ^28^ ^]^

**Figure 6:**
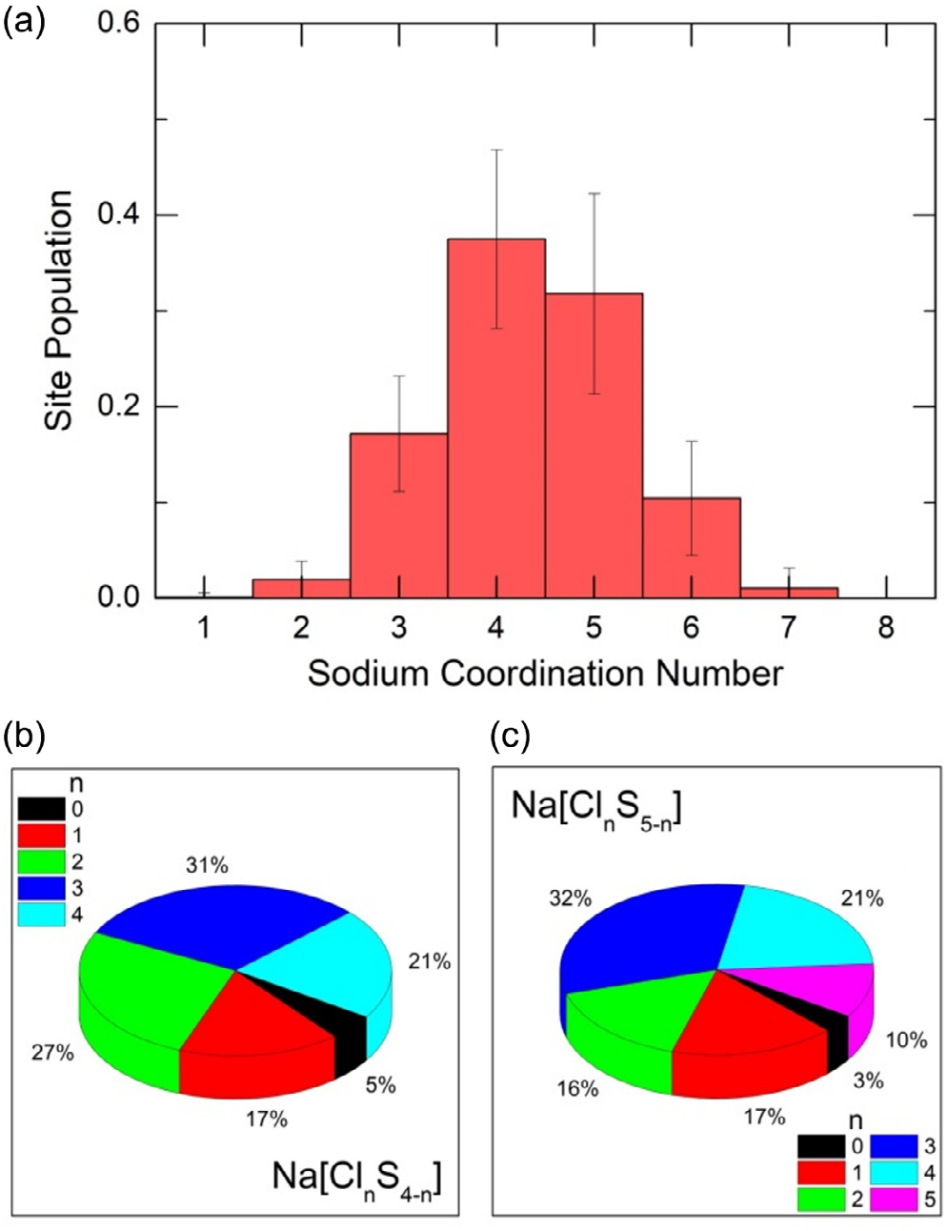
a) FPMD‐derived sodium coordination distributions in liquid 2NaCl–Ga_2_S_3_ at 1100 K; mixed sodium environment in b) $\text{Na}\left[{\text{Cl}}_{n}S_{4-n}\right]$, and (c) $\text{Na}\left[{\text{Cl}}_{n}S_{5-n}\right]$ entities.

Sodium coordination distribution reveals multiple environments (**Figure** **6**). The major contribution yields fourfold coordinated species Na_4F_−X (37 ± 9%), followed by fivefold (32 ± 10%) and trigonal (17 ± 6%) sodium. The last statistically relevant fraction is Na_6F_−X (10 ± 6%). A significant uncertainty in coordination distribution, compared to Ga and S (Figure 5), is presumably related to fast sodium dynamics. Mixed chloride and sulfide neighbor fractions for $\text{Na}\left[{\text{Cl}}_{n}S_{4-n}\right]$ and $\text{Na}\left[{\text{Cl}}_{n}S_{5-n}\right]$ species are also shown in Figure 6b,c. A negligible (4 ± 1%) and small (15 ± 5%) populations of pure sulfide ${\text{NaS}}_{4}$/${\text{NaS}}_{5}$ and chloride ${\text{NaCl}}_{4}$/${\text{NaCl}}_{5}$ surroundings should be noted; the mixed sodium environment is predominant.

### 2.4 Raman Spectroscopy and DFT Modeling of 2NaCl–Ga_2_S_3_

**Figure 7:**
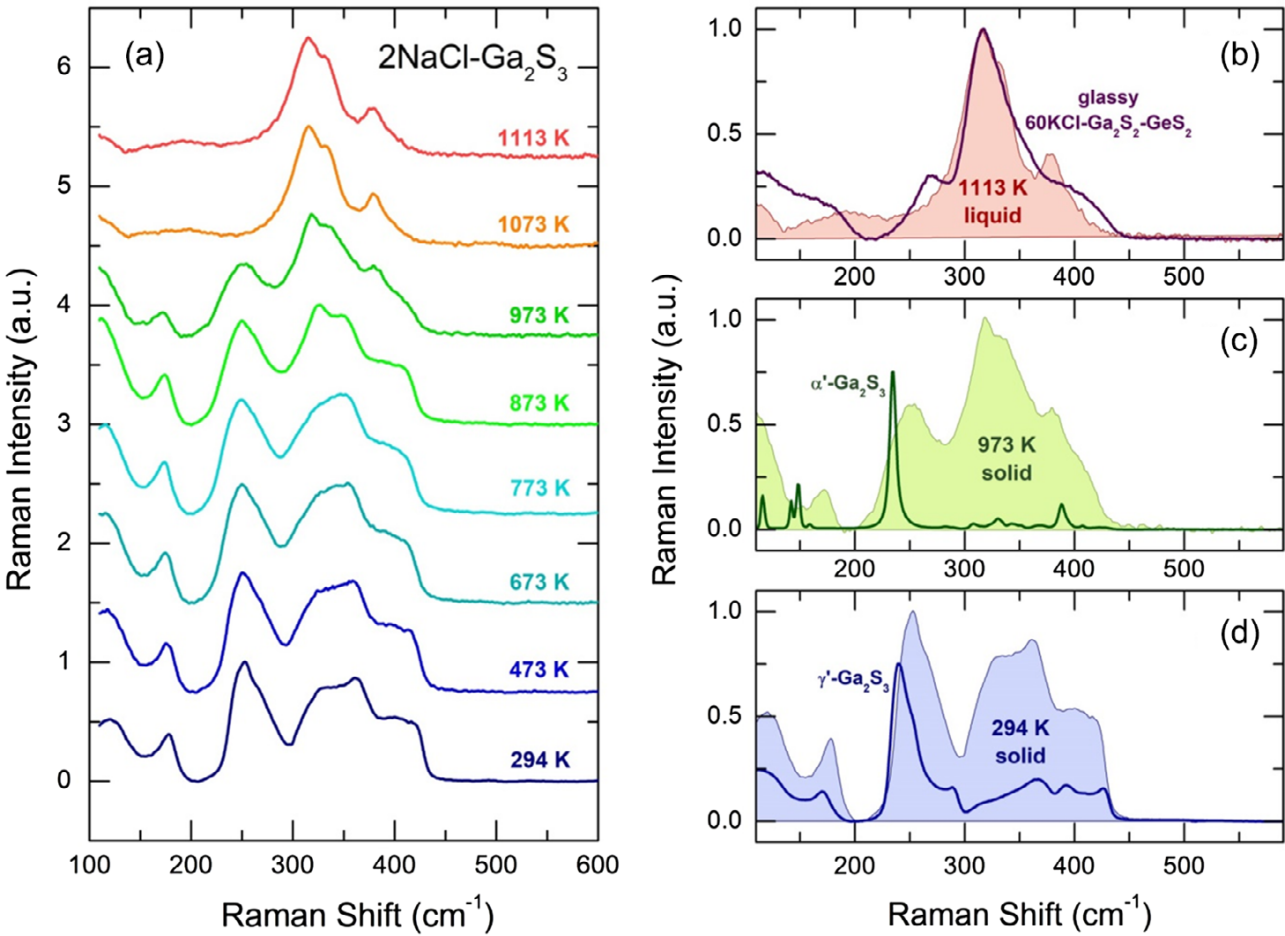
a) Raman spectra of 2NaCl**–**Ga_2_S_3_ over a wide temperature range; b) typical spectrum of normal liquid at 1113 K in comparison with glassy (KCl)_0.6_(Ga_2_S_3_)_0.32_(GeS_2_)_0.08_ at room temperature; spectra of solid 2NaCl**–**Ga_2_S_3_ at c) 973 K and d) 294 K in comparison with monoclinic α′‐Ga_2_S_3_ (space group Cc) and γ′‐Ga_2_S_3_ (space group C2/m), respectively.

The Raman spectra of 2NaCl–Ga_2_S_3_ solids and liquids over a wide temperature range from 294 to 1113 K are shown in **Figure** **7**. The Raman spectra of quenched and heated solids exhibit broad spectral features resembling those of monoclinic *γ*′‐Ga_2_S_3_ (space group C2/m), also obtained by fast quenching following NaCl and other soluble species dissolution in water.

Considering that the observed Bragg peaks only belong to sodium chloride and gallium(III) sulfide, the soluble residue should be amorphous. It is worth noting extended glass‐forming domains occur in the MCl–Ga_2_S_3_–GeS_2_ systems, where M = Li, Na, K, Rb, Cs (Figure S9, Supporting Information)^[^ ^15^, ^39^ ^]^ In addition, bulk 2MCl–Ga_2_S_3_ glasses can be easily obtained with heavy alkali chlorides RbCl and CsCl.^[^ ^15^, ^24^, ^39^ ^]^ The amorphous contribution is also visible in the X‐Ray structure factors of semicrystalline 2NaCl–Ga_2_S_3_ alloys (Figure 1e, S2 and S3, Supporting Information).

The shape of the Raman spectra of solid samples evolves with increasing temperature; however, the characteristic features remain intact, with only changes in the relative amplitude. In contrast to diffraction data, the polymorphic γ′–α′ phase transition above 853 K is hardly observable in the Raman spectra, except for a distinct decrease in intensity of the high‐frequency Ga–S stretching at ≈430 cm^−1^ and Ga–S bending at ≈170 cm^−1^, with a simultaneous appearance of A'(4)/A'(5) bending modes of α'‐Ga_2_S_3_ at ≈150 cm^−1^.^[^ ^40^, ^41^ ^]^

A drastic evolution occurs above the melting point. First, the intense low‐frequency Ga–S stretching in the vicinity of 250 cm^−1^ disappears completely. This mode originates from the A’(6) stretching^[^ ^40^, ^41^ ^]^ in monoclinic α′‐Ga_2_S_3_ and related compounds and/or nanocrystals. The remaining poorly resolved multimodal features between 260 and 440 cm^−1^ are similar to those in MCl‐rich glasses, such as (KCl)_0.6_(Ga_2_S_3_)_0.32_(GeS_2_)_0.08_, also shown in Figure 7b. In addition, a very broad feature centered at 190 cm^−1^ replaces the three peaks below 280 cm^−1^.

**Figure 8:**
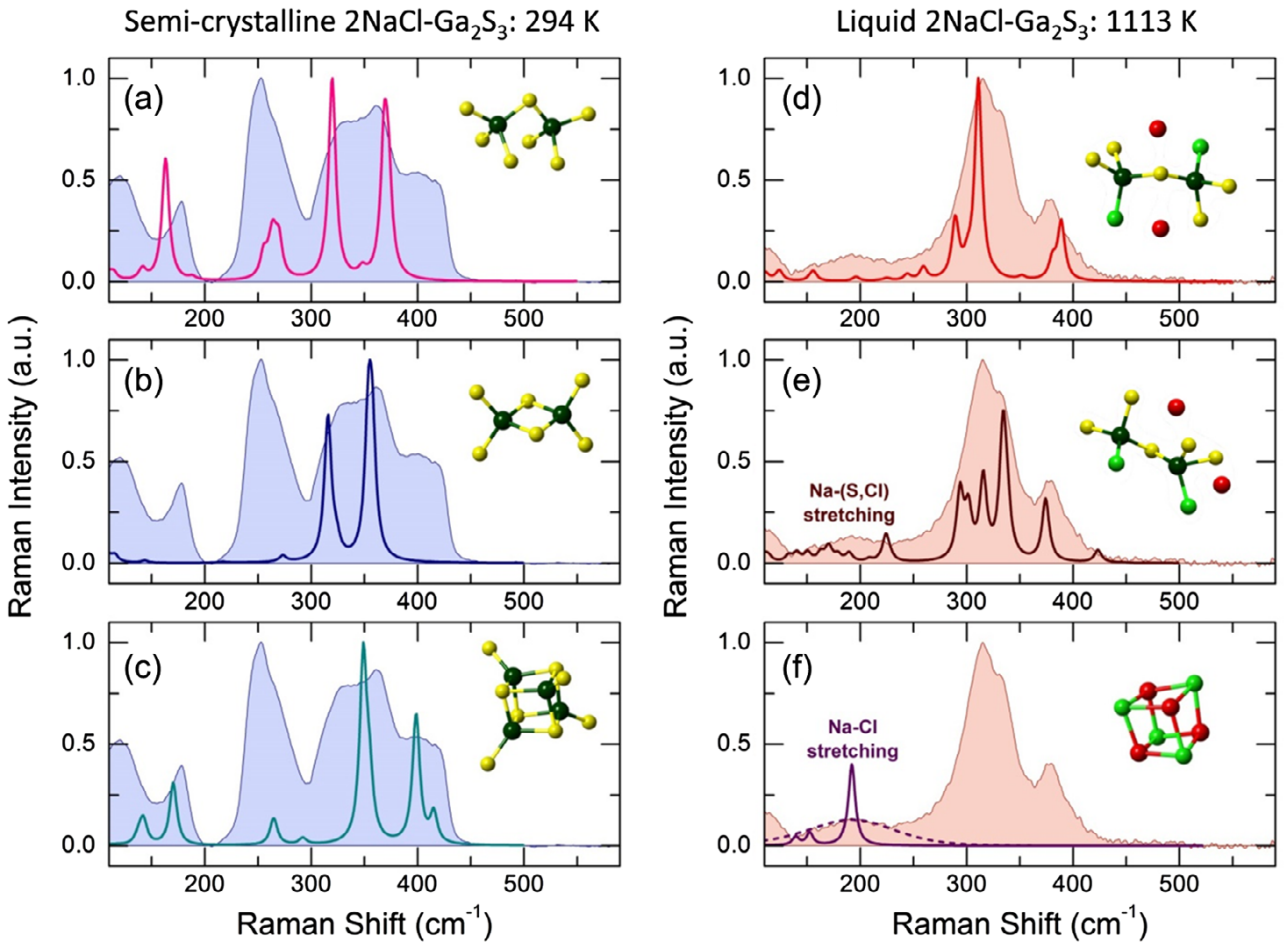
Experimental and DFT Raman spectra of a–c) semicrystalline and d–f) liquid 2NaCl–Ga_2_S_3_ at 294 K and 1113 K, respectively; and size‐limited clusters: a) CS–Ga_2_S_7_H_6_, b) ES–Ga_2_S_6_H_4_, c) Ga_4_S_8_H_4_, d) CS–Ga_2_Cl_2_S_5_Na_2_H_2_, e) CS–Ga_2_Cl_2_S_5_Na_2_H, f) Na_4_Cl_4_; the optimized clusters are shown in the insets. The terminal H species are not displayed, and the H‐related vibrations were removed from the DFT spectra. Two types of Na–Cl stretching are shown in (f): the solid line represents the vibrations of a Na_4_Cl_4_ cube of $T_{d}$ symmetry with the Na–Cl distances of 2.7 Å; the dashed line corresponds to a weighted average of the symmetric Na–Cl stretching in Na_4_Cl_4_ cubes possessing the Na–Cl bond distance distribution taken from FPMD modeling.

Density Functional Theory (DFT) modeling using size‐limited Ga–S, Ga–S–Cl, and related clusters appears to be useful to identify the observed vibrations. **Figure** **8** reveals experimental and DFT Raman spectra for two contrasting cases: semicrystalline and liquid 2NaCl–Ga_2_S_3_ at 294 and 1113 K, respectively. The DFT‐optimized clusters, derived from the FPMD simulations, consistently reproduce the characteristic vibrations. An intense bimodal broad feature of the semicrystalline sample, centered at ≈350 cm^−1^, contains symmetric and asymmetric Ga–S stretching in CS–Ga_2_S_7_ and ES–Ga_2_S_6_ dimers, involving either bridging or “terminal” sulfur species, respectively. The “terminal” sulfur atoms are, in fact, also bridging because they are bound to terminal H species, not shown in Figure 8. This bimodal feature also contains symmetric Ga–S breathing at 349 cm^−1^ of the inner cage of the cubane‐type Ga_4_S_8_ cluster, formed by fourfold coordinated Ga_4F_ and trigonal S_3F_ atoms. High‐frequency modes, centered at ≈400 cm^−1^, are associated with asymmetric (399 cm^−1^) and symmetric (415 cm^−1^) gallium−“terminal” sulfur stretching in the cubane‐like entity, which reproduces the most rigid part of gallium (III) sulfide lattice.

A multimodal aspect of the stretching feature in the stable liquid at 1113 K assumes several contributions. Mixed corner‐ and edge‐sharing dimers (Figure 8, and S10, Supporting Information) reasonably well describe the observed vibrations, consisting of symmetric and asymmetric Ga–S and Ga–Cl stretching modes between 260 and 440 cm^−1^. In particular, the most intense peak at 311 cm^−1^ (Figure 8d) corresponds to symmetric in‐phase Ga–S stretching in CS–Ga_2_Cl_2_S_5_Na_2_; the feature at 334 cm^−1^ (Figure 8e) is a combined in‐phase asymmetric Ga–Cl stretching and symmetric vibration of a Ga–S–Ga bridge in another structural isomer CS–Ga_2_Cl_2_S_5_Na_2_. A distinct mode at 380 cm^−1^ and its high‐frequency tail consists of multiple combined stretching of Ga–S(*t*), Ga–Cl, and/or Ga–S(*b*) bonds in CS and ES units, where S(*t*) and S(*b*) labels denote real terminal and bridging sulfur in Na‐containing entities. The most intense frequencies and their assignments are collected in Table S1, Supporting Information.

The emerging broad feature at 190 cm^−1^ (Figure 8e,f) is associated with Na–Cl and Na–S stretching, as it reveals vibrations of the Na_4_Cl_4_ cluster of the $T_{d}$ symmetry. A weighted average of the Na–Cl breathing frequencies in Na_4_Cl_4_ clusters, reproducing the FPMD‐derived distribution of the Na–Cl NN distances (Figure 4g), yields a broad Na–Cl stretching mode resembling experimental results. Similar Na–S stretching modes were observed in crystalline Na_2_S and vitreous Na_2_S–GeS_2_ alloys.^[^ ^31^, ^42^, ^43^ ^]^ Sodium‐containing clusters, such as CS–Ga_2_Cl_2_S_5_Na_2_ (the inset in Figure 8e), also reveal Na–Cl and Na–S vibrations in the vicinity of 200 cm^−1^; however, their intensity is smaller compared to Ga–S or Ga–Cl stretching.

Basically, the Raman data confirm a significant distinction in the short‐ and intermediate‐range structure between the solid and liquid phases of 2NaCl–Ga_2_S_3_, and the absence of crystal‐like atomic arrangements of gallium (III) sulfide above the melting temperature. These results align perfectly with the diffraction experiments and FPMD simulations.

### 2.5 Sodium Diffusion in Liquid and Vitreous 2NaCl–Ga_2_S_3_

Amazingly fast sodium diffusion was found in 2NaCl**–**Ga_2_S_3_ over a wide temperature range. Mean‐square displacements (MSD) of atomic species, $\left\langle r_{i}^{2}\left(t\right)\right\rangle$, were used to compute the diffusion coefficients $D_{i}$

(2) $$\left\langle r_{i}^{2}\left(t\right)\rangle =\langle \frac{1}{N_{i}}\left\{\sum_{i=1}^{N_{i}}{\left[r_{i}\left(t\right)-r_{i}\left(0\right)\right]}^{2}\right\}\right\rangle$$

where $r_{i}\left(t\right)$ and $r_{i}\left(0\right)$ are the positions of particle *i* at time *t* and the initial time, respectively, and $N_{i}$ represents the total number of particles in the simulation box, and the angle brackets denote the average over initial times.

**Figure 9:**
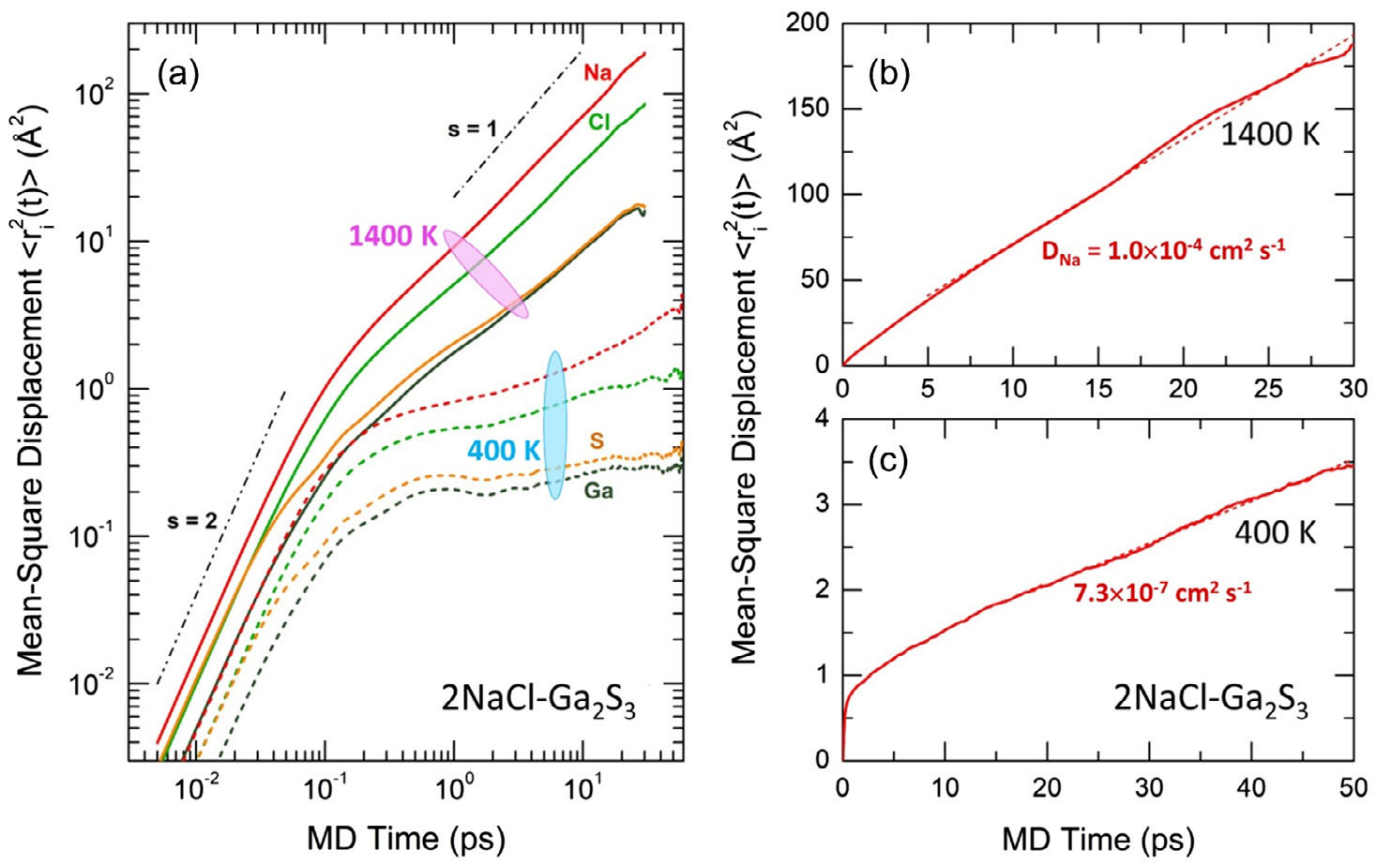
Atomic dynamics in liquid and glassy 2NaCl–Ga_2_S_3_: a) mean‐square displacements $\left\langle r_{i}^{2}\left(t\right)\right\rangle$ of Na (red), Cl (green), Ga (dark green), and S (dark yellow) at 400 and 1400 K on a log‐log scale; $\left\langle r_{\text{Na}}^{2}\left(t\right)\right\rangle$ on a linear scale at b) 1400 K and c) 400 K. The characteristic slopes *s* for the ballistic regime (*s* = 2) and diffusive behavior (*s* = 1) are presented in (a) by the dash‐dot‐dot and dashed‐dotted lines, respectively. The calculated sodium diffusion coefficients $D_{\text{Na}}$ are also indicated in (b) and (c).

Typical MSD of sodium, chlorine, gallium, and sulfur in the high‐temperature liquid (1400 K) and vitreous solid (400 K) are shown in **Figure** **9**. Below 30 fs, the ballistic regime^[^ ^44^ ^]^ is observed, $\langle r_{\text{Na}}^{2}\left(t\right)\rangle \propto t^{s}$, where the power‐law exponent *s* = 2. Above 1 ps at high temperatures, the atomic species reveal a diffusion regime, *s* = 1, with the highest sodium diffusion coefficient $D_{\text{Na}}$. Surprisingly, the chlorine MSD is only by a factor of 2 lower than $\left\langle r_{\text{Na}}^{2}\left(t\right)\right\rangle$, while $\left\langle r_{\text{Ga}}^{2}\left(t\right)\right\rangle$ and $\left\langle r_{S}^{2}\left(t\right)\right\rangle$ are smaller by one order of magnitude. With decreasing temperature, both gallium and sulfur gradually lose the diffusive motion, while sodium remains mobile up to 400 K. It should be noted that the expected glass transition temperature $T_{g}$ for 2NaCl–Ga_2_S_3_ appears to be about 480 K, extrapolating the $T_{g}$ values in the NaCl–Ga_2_S_3_–GeS_2_ glassy system as well as those for 2MCl–Ga_2_S_3_ glasses, were M = Rb, Cs.

The Einstein equation was applied to calculate the diffusion coefficients

(3) $$D_{i}=\frac{1}{6}\underset{t\to \infty }{\lim}\frac{\partial \langle r_{i}^{2}\left(t\right)\rangle }{\partial t}$$

**Figure 10:**
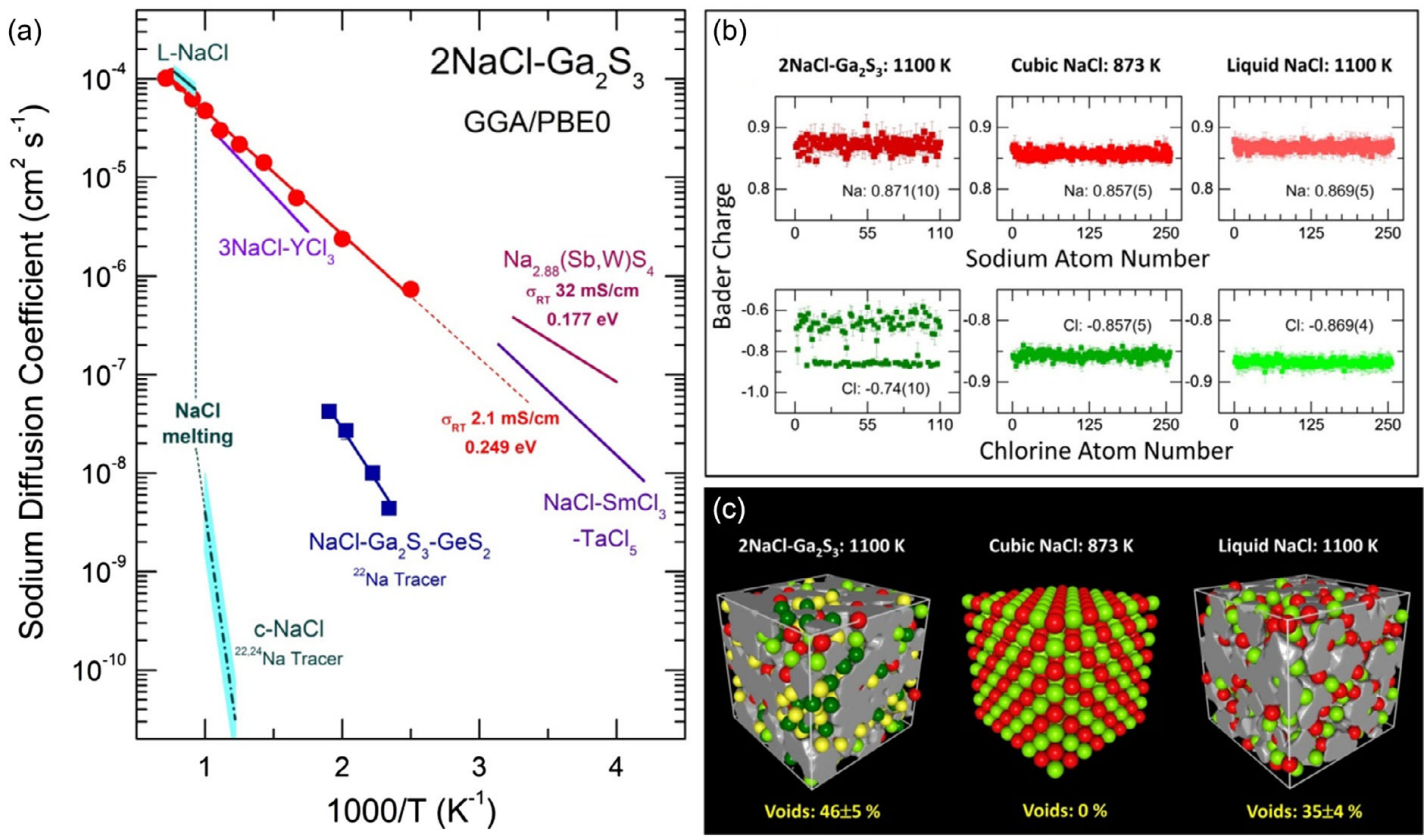
a) Experimental and calculated sodium diffusion coefficients $D_{\text{Na}}$ in liquid and glassy 2NaCl–Ga_2_S_3_ (this work, FPMD), liquid^[^ ^45^ ^]^ and solid^[^ ^48^, ^49^, ^50^, ^51^, ^52^ ^]^ sodium chloride, and glassy NaCl–Ga_2_S_3_–GeS_2_ (this work, ^22^Na tracer diffusion). Sodium diffusion coefficients for liquid 3NaCl–YCl_3_ (FPMD),^[^ ^47^ ^]^ and solid Na^+^ superionic conductors Na_2.88_(Sb,W)S_4_ and NaCl–SmCl_3_–TaCl_5_, recalculated from the ionic conductivity,^[^ ^10^, ^53^ ^]^ are also plotted. b) Bader charges and c) microscopic voids in liquid 2NaCl–Ga_2_S_3_, cubic and liquid NaCl. Further details can be found in the text.

The derived sodium diffusivity $D_{\text{Na}}$ in normal and supercooled liquid, and vitreous 2NaCl–Ga_2_S_3_ are shown in **Figure** **10** together with that in liquid NaCl, calculated from the ionic conductivity $\sigma_{i}$ data^[^ ^45^ ^]^ using the Nernst–Einstein relation

(4) $$D_{\sigma }=\frac{k_{B}T}{{\left(Ze\right)}^{2}}x^{-1}\sigma_{i}$$

where $D_{\sigma }$ is the diffusion (conductivity) coefficient, Ze the electric charge of the carrier ion, *x* the mobile ion concentration, and $k_{B}$ and *T* are the Boltzmann constant and temperature, respectively. The Haven ratio^[^ ^46^ ^]^ $H_{R}=(D_{\text{Na}}+D_{\text{Cl}})/D_{\sigma }$ was assumed to be $H_{R}=1$ and $D_{\text{Na}}=1.5D_{\text{Cl}}$.

We note a close similarity between the sodium diffusion coefficients in the two liquids (2NaCl–Ga_2_S_3_ and NaCl), as well as in a promising Na^+^ halide ion conductor 3NaCl–YCl_3_ in the liquid state.^[^ ^47^ ^]^ In contrast to sodium chloride, whose ionic conductivity and the ^22^Na and/or ^24^Na tracer diffusion decreases by four orders of magnitude below the melting point,^[^ ^48^, ^49^, ^50^, ^51^, ^52^ ^]^ supercooled and glassy 2NaCl–Ga_2_S_3_ follows Arrhenius behavior up to 400 K. Additionally, the extrapolation to 298 K yields the room‐temperature conductivity $\sigma_{\text{RT}}$ = 2.1 mS cm^−1^ with the activation energy of 0.249 eV. The derived $\sigma_{\text{RT}}$ is only by a factor of 15 lower than that in one of the best sodium sulfide superionic conductors, i.e., Na_2.88_(Sb,W)S_4_ (32 mS cm^−1^ and 0.177 eV),^[^ ^53^ ^]^ and comparable with the Na^+^ conductivity (2.7 mS cm^−1^ and 0.26 eV) and sodium diffusion in the record‐holding hybrid sodium chloride solid electrolyte (Na_0.75_Sm_1.75_Cl_6_)_0.62_(NaTaCl_6_)_0.38_.^[^ ^10^ ^]^

In order to verify whether the FPMD‐derived sodium ion transport in vitreous 2NaCl–Ga_2_S_3_ appears to be reasonable, we undertook sodium tracer diffusion measurements for similar sulfochloride glasses, NaCl–Ga_2_S_3_–GeS_2_, which exhibit good chemical and thermal stability.^[^ ^11^ ^]^ The experimental ^22^Na tracer diffusion coefficients for a (NaCl)_0.2_(Ga_2_S_3_)_0.16_(GeS_2_)_0.64_ alloy over the temperature range from 427 to 525 K are shown in Figure 10, while the diffusion profiles and further details of the tracer measurements are given in the Supporting Information (Figure S11, Supporting Information and the related text). The obtained sodium diffusion is lower for this NaCl‐poor glass (6.41 at% Na) compared to the more concentrated vitreous 2NaCl–Ga_2_S_3_ (22.22 at% Na) but much higher compared to cubic NaCl reference, $D_{\text{Na}}\left(\text{glass}\right)/D_{\text{Na}}\left(\text{c‐NaCl}\right)$ ≳ 10^10^! The diffusion activation energy, 0.44 ± 0.05 eV, and the Haven ratio, $H_{R}$ = 0.32 ± 0.07, are also consistent with comparable Na^+^ and Ag^+^ conducting vitreous alloys.^[^ ^31^, ^54^ ^]^ In addition, the extrapolation of ionic conductivity and ^22^Na tracer diffusion parameters of NaCl–Ga_2_S_3_–GeS_2_ glasses yields the expected results for glassy 2NaCl–Ga_2_S_3_ (Figure S12, Supporting Information). Consequently, the FPMD‐derived ion transport appears to be coherent with available experimental data. Our preliminary first‐principles modeling of liquid NaCl also yields sodium and chlorine diffusion coefficients consistent with the reported conductivity data^[^ ^45^ ^]^ (Figure S13, Supporting Information).

The question arises: what is the origin of high Na^+^ ion conductivity in 2NaCl–Ga_2_S_3_? Two possible reasons can be considered: 1) available empty space for sodium migration, and 2) low barriers for sodium mobility.

The available empty space can be determined by calculating the fraction of microscopic voids and cavities in 2NaCl–Ga_2_S_3_ using the Dirichlet–Voronoi tessellation method.^[^ ^55^ ^]^ Typical snapshots of the simulation boxes with derived voids are shown in Figure 10c. The relative volume of voids normalized to the box size appears to be quite large, $V_{c}/V_{0}$ = 46 ± 5%, for liquid 2NaCl**–**Ga_2_S_3_, ensuring enough space for ion migration and high mobility. A similar $V_{c}/V_{0}$ = 35 ± 4% value was found in molten NaCl, which exhibits comparable sodium diffusion. In contrast, in crystalline sodium chloride, there are no microscopic voids if the radius of the test particle is above 2.5 Å (in our calculations, the radius was taken as 2.7 Å, corresponding to the Na–Cl interatomic distance in NaCl and 2NaCl–Ga_2_S_3_ liquids, as recommended for the Dirichlet–Voronoi method^[^ ^55^ ^]^). This last result aligns with a drastic drop in sodium diffusion of four orders of magnitude for cubic sodium chloride just below the melting point.

The Bader charges^[^ ^56^ ^]^ on sodium, chlorine, and sulfur reflect electrostatic interactions between sodium cations and anions, and could affect the mobility barriers. Previously, energy landscapes with low barriers for alkali ion migration were derived for lithium and sodium halide superionic conductors,^[^ ^5^, ^6^, ^7^, ^57^ ^]^ as well as intrinsic frustration in the chemical bond dynamics, leading to increased cation mobility.^[^ ^58^ ^]^

The calculated sodium $q_{\text{Na}}$ and chlorine $q_{\text{Cl}}$ Bader charges for liquid 2NaCl–Ga_2_S_3_, crystalline, and molten sodium chloride are shown in Figure 10b. The data for cubic NaCl are consistent with the reported results:^[^ ^59^ ^]^ $q_{\text{Na}}$ = + 0.857 ± 0.005 and $q_{\text{Cl}}$ = −0.857 ± 0.005, and slightly lower than those in the NaCl melt: +0.869 ± 0.005 and −0.869 ± 0.004, respectively. The sodium charge in liquid 2NaCl–Ga_2_S_3_, $q_{\text{Na}}$ = + 0.871 ± 0.010, is very similar to that in molten sodium chloride, suggesting that chlorine and sulfur species in the vicinity of sodium have similar charges, confirmed by the $q_{S}$ values (Figure S14, Supporting Information). In contrast, the chlorine charges in 2NaCl–Ga_2_S_3_ exhibit two different populations: chlorine connected to sodium reveals $q_{\text{Cl}\left(\text{Na}\right)}$ = −0.861 ± 0.005 while $q_{\text{Cl}\left(\text{Ga}\right)}$ = −0.653 ± 0.017. The Bader charges on gallium and chlorine in gallium trichloride liquid at 400 K (Figure S15, Supporting Information) are consistent with this result.

The derived Bader charges appear to be sensitive to variations in electronic density in the vicinity of specific atom species but seem to be unaffected by the ionic mobility of NNs and vice versa. Nevertheless, a lower average Bader charge $q_{\text{Cl}}$ = −0.74 in isomeric 2NaCl–Ga_2_S_3_ may lead to lower barriers in the presumably saddle‐shaped energy landscape near Ga–Cl sites, facilitating faster Na^+^ migration. Additionally, the magnitude of empty space nicely reflects trends in fast ion transport.

### 2.6 Rigidity‐Driven Isomerism as a Searching Strategy for Fast Vitreous Sodium Halide Conductors

The excessive rigidity of crystalline Ga_2_S_3_ is caused by tetrahedral gallium coordination and a significant fraction of sulfur triclusters S_3F_ (constituting two‐thirds of all sulfur species). Consequently, the average local coordination in c‐Ga_2_S_3_ is *r* = 3.2. The excessively rigid crystalline gallium (III) sulfide does not survive the viscous flow, breaking up on a local scale. Raman spectroscopy provides a clear illustration of this process near the melting point, between 873 and 1073 K (see Figure 7a). The disappearance of the high‐frequency Ga–S stretching feature at 400 ≲ *ω* ≲ 430 cm^−1^ and the decrease in vibrational intensity at around 350 cm^−1^ above the eutectic temperature are associated with a sudden reduction in the population of sulfur triclusters. The S_3F_ containing units are represented by the cubane‐type Ga_4_S_8_ entity in DFT modeling (Figure 8c). This reduction is also confirmed by FPMD. The fraction of S_3F_ species in liquid 2NaCl–Ga_2_S_3_ at 1100 K is only 26 ± 3% (Figure 5a) instead of 67%. The diminished S–Ga atomic pairs are substituted by Cl–Ga contacts while maintaining tetrahedral Ga coordination intact. These structural changes lead to a much less constrained gallium subnetwork with an average coordination number *r* = 2.53. The derived average coordination in the stable 2NaCl–Ga_2_S_3_ liquid appears to be close to the optimally constrained disordered network with *r* = 2.4,^[^ ^60^, ^61^, ^62^ ^]^ originating from the Maxwell–Calladine index theorem.^[^ ^63^, ^64^, ^65^, ^66^ ^]^

The appearance of new partners (chlorine for gallium and sulfur for sodium) in the stable liquid signifies the structural isomerism in the NaCl–Ga_2_S_3_ system, which is fully reversible upon crystallization. The emerging structural isomers in the stable liquid are significantly less dense compared to gallium (III) sulfide and sodium chloride binaries (Figure S16, Supporting Information). The additional empty space is favorable for fast Na^+^ transport in the liquid but, more importantly, can be frozen in a metastable glassy isomer with a mixed gallium and sodium environment, as revealed by our FPMD simulations and experimental reports on alkali halide gallium sulfide glasses.^[^ ^15^, ^16^, ^17^ ^]^

The FPMD‐predicted high Na^+^ conductivity may be realized in a series of sodium halide glasses formed by chalcogenides of Group 13 elements (Al, Ga, In), which exhibit similar rigid crystal lattices as Ga_2_S_3_ and, therefore, are potential candidates for rigidity‐driven structural isomerism. Thallium chalcogenides and telluride systems seem to be exceptions to this assumption. Fully vitreous alloys were not reported for sodium halide–A_2_X_3_ systems, where A = Al, Ga, In and X = S, Se. Small additions of Group 14 chalcogenides (Si, Ge, Sn) might be beneficial for improving the glass‐forming ability while presumably keeping high Na^+^ conductivity intact.

## 3 Conclusions

A rare phenomenon—phase‐dependent chemical interactions between binary components—was investigated for stable crystals and liquids in the eutectic system NaCl–Ga_2_S_3_ using diffraction and Raman spectroscopy over a wide temperature range from 294 to 1223 K, supported by first‐principles simulations. In the crystalline state of a nearly eutectic composition 2NaCl–Ga_2_S_3_, both sodium chloride and gallium (III) sulfide remain chemically intact in accordance with the phase diagram. The only change was associated with a polymorphic transition from cubic γ‐Ga_2_S_3_ to the monoclinic α'‐form above 853 K, occurring in the same temperature range as for pure Ga_2_S_3_.

In contrast, the combined use of pulsed ND and high‐energy X‐ray scattering reveals strong chemical interactions in the stable 2NaCl–Ga_2_S_3_ liquid, where gallium and sodium species become connected to new partners, chlorine and sulfur, respectively, forming structural isomers of the crystalline binaries. This process is fully reversible; both slow cooling or fast quenching of molten 2NaCl–Ga_2_S_3_ yield polycrystalline mixtures of either NaCl/α'‐Ga_2_S_3_ or NaCl/γ'‐Ga_2_S_3_, where γ'‐Ga_2_S_3_ is a distorted monoclinic polymorph (space group C2/m) of the cubic γ‐Ga_2_S_3_ form.

The rigidity paradigm appears to be the driving force behind the observed phenomenon. The excessively rigid crystalline Ga_2_S_3_, composed of tetrahedral gallium species and threefold coordinated sulfur triclusters S_3F_ (constituting two‐thirds of all sulfur species), with the average local coordination number *r* = 3.2, does not survive the viscous flow and breaks up on a local scale. Raman spectroscopy measurements below and above the melting point, supported by DFT modeling of vibrational properties, demonstrate a drastic reduction in the population of sulfur triclusters in both eutectic and normal NaCl–Ga_2_S_3_ liquids. FPMD confirms this finding, revealing a strongly diminished S_3F_ fraction, 26 ± 3%, and a reduced *r* = 2.53. This derived average coordination appears to be close to the optimally constrained disordered network with *r* = 2.4.

Unexpectedly, the FPMD simulations predict fast sodium diffusion in the stable and supercooled 2NaCl–Ga_2_S_3_ liquid, as well as in a frozen isomeric glass, comparable with the best sodium halide superionic conductors. The conductivity reaches 2.1 mS cm^−1^ at room temperature with an activation energy of 0.249 eV. Experimental ^22^Na tracer diffusion measurements on similar sulfochloride glasses with lower NaCl content have shown the sodium diffusion coefficients higher than those in crystalline NaCl by ten orders of magnitude. Extrapolating these values to the 2NaCl–Ga_2_S_3_ composition yields the FPMD‐predicted sodium diffusion and Na^+^ conductivity parameters. A significant fraction of empty space in the disordered lattice, resulting from the removal of rigidity constraints and dense packing, as calculated using Dirichlet–Voronoi tessellation, is the origin of high sodium diffusivity. Consequently, the rigidity‐driven structural isomerism open up a searching strategy for the discovery of novel glassy sodium halide fast ion conductors, promising for solid‐state batteries and energy applications.

The outstanding question in this context remains the experimental verification of the searching strategy, which involves synthesizing sodium halide‐rich structural isomers in NaY–A_2_X_3_–BX_2_ glassy systems, where Y = Cl, Br, I; A = Ga, In; B = Ge, Sn; and X = S, Se. These isomers are expected to exhibit high sodium conductivity (about 1 mS cm^−1^), good chemical stability, and wide electrochemical window, advancing R&D in the field of efficient high‐density solid‐state batteries.

## 4 Experimental Section

4.1

4.1.1

##### Synthesis

Chalcohalide compositions in the NaCl–Ga_2_S_3_ and NaCl–Ga_2_S_3_–GeS_2_ systems were prepared by classical melt quenching from high purity elements (Ga, Ge, and S, 99.999%, Neyco or Acros Organics) and sodium chloride (99.85%, Acros Organics). The mixtures were sealed in silica tubes under vacuum (10^−4^ Pa). The batches were heated at a rate of 1 K min^−1^ to 1200 K, homogenized at this temperature for a few days, and then quenched in cold water. The 2NaCl–Ga_2_S_3_ samples for HE‐XRD measurements after primary synthesis were placed into thin‐walled silica tubes (ID 2 mm, OD 3 mm), evacuated and sealed under vacuum.

##### Diffraction Measurements over a Wide Temperature Range

Time‐of‐flight ND experiments have been carried out at the ISIS spallation neutron source (Rutherford‐Appleton Laboratory, UK) using the GEM diffractometer. HE‐XRD experiments of crystalline and liquid 2NaCl–Ga_2_S_3_ over a wide temperature range from 300 to 1223 K were conducted at the BL04B2 beamline of the Spring‐8 facility (Hyogo prefecture, Japan). Two separate experiments were performed using X‐ray energies of 61.350 and 61.199 keV, providing data at *Q* values up to 25 Å^−1^ in a 1D scanning mode with a detector array consisting of a Ge diode and three CdTe detectors. In addition to the diffraction experiments, separate transmission measurements of molten 2NaCl–Ga_2_S_3_ were carried out using an ionization chamber. The measured X‐ray absorption as a function of temperature was used to calculate the *T*‐dependent density of liquid 2NaCl–Ga_2_S_3_. Additional HE‐XRD measurements of slowly cooled (with a furnace) and rapidly quenched 2NaCl–Ga_2_S_3_ (in icy water from 1223 K) were conducted at the 6‐ID‐D beamline of the Advanced Photon Source (Argonne National Laboratory, Lemont, IL, USA). The photon energy used was 100.398 keV, with a corresponding wavelength of 0.123493 Å. Further experimental details and data analysis can be found in the Supporting Information.

##### XRD and LeBail Refinement

XRD pattern of crystalline Ga_2_S_3_ samples were recorded at room temperature using a Bruker D8 A25 diffractometer equipped with a copper anode (λ = 1.5418 Å), operating at 40 kV and 40 mA, in Bragg–Brentano reflection geometry. A 1D position‐sensitive detector, Bruker LynxEye XE‐T, covering 3° in 192 channels was used. Phase identification was conducted with Bruker EVA 6.1 software coupled to PDF2023 database.^[^ ^67^ ^]^ Full powder pattern matching was done by LeBail extraction, using the JANA2020 software.

##### Raman Spectroscopy Measurements

A LabRam HR microRaman spectrometer (Jobin Yvon Horiba Group) was used for the measurements at room temperature. Raman scattering was excited by a 785 nm solid‐state laser and recorded in the 50–850 cm^−1^ spectral range. The laser power was 4 mW. Two to three spectra were registered for each sample at different positions to verify the sample homogeneity and the absence of photoinduced phenomena. Raman spectra over the temperature range 294 ≤ *T* ≤ 1113 K were measured using a Senterra Raman spectrometer (Bruker) equipped with a microscope and a Linkam TS1000 hot stage. The spectra were excited by a 785 nm laser diode with a power of 10 mW and recorded in the 75–1500 cm^−1^ spectral range (reliable data above 100 cm^−1^). The 2NaCl–Ga_2_S_3_ sample was placed in a silica tube (2 mm ID/3 mm OD, length 25 mm) and sealed under vacuum.

##### Sodium Tracer Diffusion Measurements

The ^22^Na tracer (the life‐time $t_{1/2}$ = 2.6027 years, iThemba LABS, Faure, South Africa, radionuclide purity 99.9%) was used for tracer diffusion experiments in a thin‐layer geometry. The diffusion anneals in a furnace over the temperature range from 408 to 525 K were from 2 to 39 days and terminated by quenching the samples in air. A high‐purity Ge detector GX1018 and LYNX gamma spectrometer (Canberra Ind., USA) was used to measure the initial and residual activity of the sample before and after sectioning. The spectrometer calibration in the energy range from 20 to 1600 keV was carried out using ^109^Cd, ^152^Eu, and ^241^Am sources with different activities. The gamma activity of the samples was determined using two characteristic ^22^Na photopeaks at 511 and 1275 keV. Further experimental details are provided in the Supporting Information.

##### First‐Principles Simulations

The DFT calculations of vibrational spectra were carried out using Gaussian 16 software. The structural optimization and harmonic vibrational frequency calculations were performed for size‐limited clusters: CS–Ga_2_S_7_H_6_, ES–Ga_2_S_6_H_4_, Ga_4_S_8_H4, CS–Ga_2_Cl_2_S_5_Na_2_H_2_, ES–Ga_2_Cl_2_S_4_Na_2_, Na_4_Cl_4_, etc. The Becke three‐parameter hybrid exchange functional and the Lee–Yang–Parr correlation functional (B3LYP)^[^ ^68^, ^69^ ^]^ were applied for these simulations. The small‐core relativistic pseudopotential basis set (cc‐pVTZ‐PP)^[^ ^70^ ^]^ and the effective core potentials^[^ ^71^ ^]^ were used for cluster geometry optimization and Raman intensity calculations. Most of the structures were optimized using the tight convergence option ensuring adequate convergence and reliability of computed wavenumbers. An extra quadratically convergent self‐consistent field procedure^[^ ^72^ ^]^ was employed for difficult convergence cases.

Modeling of the diffraction data was carried out using Born–Oppenheimer molecular dynamics implemented within the CP2K package. The GGA^[^ ^32^ ^]^ and the PBE0 hybrid exchange–correlation functional^[^ ^33^, ^34^ ^]^ combining the exact Hartree–Fock and DFT approaches were used, providing better agreement with experiment. The Grimme dispersion corrections D3BJ^[^ ^73^ ^]^ were also employed. The initial atomic configurations for liquid 2NaCl–Ga_2_S_3_ were created and optimized using the RMC_POT++ code^[^ ^74^ ^]^ against the experimental X‐ray and neutron data. The size of the cubic simulation box, containing 495 atoms (110 Na, 110 Cl, 110 Ga, and 165 S), was chosen to match the experimental density. Further optimization was carried out using DFT, applying the molecularly optimized correlation consistent polarized triple‐zeta valence basis set along with the norm‐conserving relativistic Goedecker–Teter–Hutter‐type pseudopotentials.^[^ ^75^ ^]^ FPMD simulations were performed using a canonical NVT ensemble with a Nosé–Hoover thermostat. The pyMolDyn code^[^ ^55^ ^]^ applying the Dirichlet–Voronoi tessellation was used for the calculation of microscopic voids and cavities. Electron density distribution was collected at the same level of theory and stored in Gaussian CUBE file format. The Bader atomic charges were calculated using the algorithm for doing analysis on a charge density grid.^[^ ^57^ ^]^ The derived charges were averaged over 1 ps for all atoms of the simulation box. Further simulation details can be found in the Supporting Information.

## Conflict of Interest

The authors declare no conflict of interest.

## Author Contributions

**Maria Bokova**: synthesis; Raman spectroscopy; DFT simulations. **Mohammad Kassem**: synthesis; Raman spectroscopy. **Takeshi Usuki**: HE‐XRD over a wide T‐range and data analysis. **Andrey Tverjanovich**: Raman spectroscopy measurements over a wide T‐range. **Anton Sokolov**: FPMD simulations, network statistics and Bader analysis. **Daniele Fontanari**: FPMD simulations, network statistics and Bader analysis. **Alex C. Hannon**: ND and data analysis. **Chris J. Benmore**: HE‐XRD and PDF analysis. **Igor Alekseev**: tracer diffusion measurements and data analysis. **Shinji Kohara**: HE‐XRD over a wide T‐range and data analysis. **Pascal Roussel**: XRD and LeBail refinement. **Maxim Khomenko**: FPMD simulations, network statistics and Bader analysis. **Koji Ohara**: HE‐XRD over a wide T‐range and data analysis. **Yohei Onodera**: HE‐XRD over a wide T‐range and data analysis. **Arnaud Cuisset**: DFT simulations. **Eugene Bychkov**: Conceptualization; ND and data analysis; paper writing with the contributions of all authors. All authors have given approval to the final version of the manuscript.

## Supporting information

Supplementary Material

## Data Availability

The data that support the findings of this study are available from the corresponding author upon reasonable request.
