# Supplementary material for: Rigidity‐Driven Structural Isomers in the NaCl–Ga2S3 System: Implications for Energy Storage
Source: Small Sci. 2024 Oct 1;5(1):2400371. doi: 10.1002/smsc.202400371 (PMC11935002; doi:10.1002/smsc.202400371)
Supplement: Supplementary file 1 — Supplementary Material [file SMSC-5-2400371-s001.pdf]

SUPPORTING INFORMATION

Rigidity-Driven Structural Isomers in the NaCl-Ga_2S_3 System: Implications for Energy Storage

Maria Bokova, Mohammad Kassem, Takeshi Usuki, Andrey Tverjanovich, Anton Sokolov, Daniele Fontanari, Alex C. Hannon, Chris J. Benmore, Igor Alekseev, Shinji Kohara, Pascal Roussel, Maxim Khomenko, Koji Ohara, Yohei Onodera, Arnaud Cuisset, and Eugene Bychkov*

M. Bokova, M. Kassem, A. Sokolov, D. Fontanari, I. Alekseev, A. Cuisset, E. Bychkov
Laboratoire de Physico-Chimie de l'Atmosphère, Université du Littoral Côte d'Opale, 59140 Dunkerque, France
Email: eugene.bychkov@univ-littoral.fr

T. Usuki
Faculty of Science, Yamagata University, Yamagata 990-8560, Japan

A. Tverjanovich
Institute of Chemistry, St. Petersburg State University, 198504 St. Petersburg, Russia

A. C. Hannon
ISIS Facility, Rutherford Appleton Laboratory, Chilton, Didcot OX11 0QX, U.K.

C. J. Benmore
X-ray Science Division, Advanced Photon Source, Argonne National Laboratory, Lemont, Illinois 60439, United States

S. Kohara, Y. Onodera
Quantum Beam Diffraction Group, Center for Basic Research on Materials, National Institute for Materials Science, 1-1-1 Kouto, Sayo-cho, Sayo-gun, Hyogo 679-5148, Japan

P. Roussel
Unité de Catalyse et de Chimie du Solide (UCCS), Université de Lille, CNRS, Centrale Lille, Université d'Artois, Lille, France

M. Khomenko
Faculty of Physics, Lomonosov Moscow State University, 119991 Moscow, Russia

K. Ohara
Faculty of Materials for Energy, Shimane University, 1060, Nishi-Kawatsu-Cho, Matsue, Shimane 690-8504, Japan

EXPERIMENTAL AND SIMULATION DETAILS

Synthesis

Chalcohalide compositions in the NaCl-Ga_2S_3 and NaCl-Ga_2S_3-GeS_2 systems were prepared by classical melt quenching from high purity elements (Ga, Ge and S, 99.999%, Neyco or Acros Organics) and sodium chloride (99.85%, Acros Organics). The mixtures were sealed in silica tubes under vacuum (10^{-4} Pa). The batches were heated at a rate of 1 K min^{-1} to 1200 K, homogenized at this temperature for a few days, and then quenched in cold water. The 2NaCl-Ga_2S_3 samples for high-energy X-ray diffraction measurements after primary synthesis were placed into thin-walled silica tubes (ID 2 mm, OD 3 mm), evacuated and sealed under vacuum.

Diffraction measurements over a wide temperature range

Time-of-flight neutron diffraction experiments have been carried out at the ISIS spallation neutron source (Rutherford-Appleton Laboratory, UK) using the GEM diffractometer.[s1] The sample in a sealed silica tube was placed into a vanadium container and then into a vanadium furnace for high-temperature measurements at 1073 K. An empty furnace and vanadium standard at room temperature, along with an empty silica tube at 1073 K, were also measured for calibration and background subtraction. The neutron diffraction data were corrected[s2] for background and container scattering, self-attenuation, multiple scattering, and inelasticity (Placzsek) effects to obtain the total neutron structure factor $S_{\mathrm{N}}(Q)$. The available Q-range was reduced to 30 Å^{-1} for the Fourier transform caused by a featureless $S_{\mathrm{N}}(Q)$ at Q > 30 Å^{-1} and insufficient signal-to-noise ratio.

High-energy X-ray diffraction experiments of crystalline and liquid 2NaCl-Ga_2S_3 over a wide temperature range from 300 to 1223 K were conducted at the BL04B2 beamline[s3] of the Spring-8 facility (Hyogo prefecture, Japan). Two separate experiments were performed using X-ray energies of 61.350 and 61.199 keV, providing data at Q values up to 25 Å^{-1} in a one-dimensional scanning mode with a detector array consisting of a Ge diode and three CdTe detectors. An empty silica tube was also measured at different temperatures and used for background intensity subtraction. Further data analysis included absorption, Compton scattering, and polarization corrections using standard procedures[s4] giving the total X-ray structure factor $S_{\mathrm{X}}(Q)$. In addition to the diffraction experiments, separate transmission measurements of molten 2NaCl-Ga_2S_3 were carried out using an ionization chamber. The measured X-ray absorption as a function of temperature was used to calculate the T-dependent density of liquid 2NaCl-Ga_2S_3.

Additional high-energy X-ray diffraction measurements of slowly cooled (with a furnace) and rapidly quenched 2NaCl-Ga_2S_3 (in icy water from 1223 K) were conducted at the 6-ID-D beamline of the Advanced Photon Source (Argonne National Laboratory, Lemont, IL, USA). The photon energy used was 100.398 keV, with a corresponding wavelength of 0.123493 Å. A two-dimensional (2D) setup was used for data collection with a Varex area detector, 2880 × 2880 pixels, and a pixel size of 150 × 150 μm^2. The sample-to-detector distance was 293.3 mm. The samples were measured in thin-walled silica tubes, fixed using a sample holder of the instrument. Cerium dioxide CeO_2 was used as a calibrant. The 2D diffraction patterns were reduced using the Fit2D software.[s5] The measured background intensity of the empty silica

tube was subtracted, and corrections were applied for different detector geometries and efficiencies, sample self-attenuation, and Compton scattering using standard procedures,[s6] providing the X-ray structure factor $S_X(Q)$.

X-ray Diffraction and LeBail Refinement

X-ray diffraction pattern of crystalline Ga_2S_3 samples were recorded at room temperature using a Bruker D8 A25 diffractometer equipped with a copper anode (λ = 1.5418 Å), operating at 40 kV and 40 mA, in Bragg-Brentano reflection geometry. A 1D position-sensitive detector, Bruker LynxEye XE-T, covering 3° in 192 channels was used. Phase identification was conducted with Bruker EVA 6.1 software coupled to PDF2023 database.[s7] Full powder pattern matching was done by LeBail extraction, using the JANA2020 software.[s8]

Raman Spectroscopy Measurements

A LabRam HR microRaman spectrometer (Jobin Yvon Horiba Group) was used for the measurements at room temperature. Raman scattering was excited by a 785 nm solid-state laser and recorded in the 50–850 cm^{-1} spectral range. The laser power was 4 mW. Two to three spectra were registered for each sample at different positions to verify the sample homogeneity and the absence of photoinduced phenomena.

Raman spectra over the temperature range $294 \le T \le 1113$ K were measured using a Senterra Raman spectrometer (Bruker) equipped with a microscope and a Linkam TS1000 hot stage. The spectra were excited by a 785 nm laser diode with a power of 10 mW and recorded in the 75–1500 cm^{-1} spectral range (reliable data above 100 cm^{-1}). The 2NaCl-Ga_2S_3 sample was placed in a silica tube (2 mm ID/3 mm OD, length 25 mm) and sealed under vacuum.

Sodium Tracer Diffusion Measurements

The ^{22}Na tracer (the life-time $t_{1/2}$ = 2.6027 years, iThemba LABS, Faure, South Africa, radionuclide purity 99.9%) was used for tracer diffusion experiments in a thin-layer geometry. A drop of radioactive $^{22}NaCl/HCl$ solution (pH 2) was deposited onto one face of the plane-parallel sample, kept there for 25–30 min (a typical time for isotopic sodium exchange and sorption), wiped with a filter paper, washed twice with a distilled water and then ethyl alcohol, and dried. The sample was wrapped in aluminum foil, evacuated, and sealed in a Pyrex tube at ≈1 Pa. The diffusion anneals in a furnace over the temperature range from 408 to 525 K were from 2 to 39 days and terminated by quenching the samples in air. The sides of the sample parallel to the diffusion direction were ground to eliminate surface diffusion effects. The sample was then sectioned on a parallel grinder. The thickness of each section was determined either from the density, cross-sectional area, and weight change of the sample or by direct thickness measurements. A high-purity Ge detector GX1018 and LYNX gamma-spectrometer (Canberra Ind., USA) was used to measure the initial and residual activity of the sample before and after sectioning. The spectrometer calibration in the energy range from 20 to 1600 keV was carried out using ^{109}Cd, ^{152}Eu, and ^{241}Am sources with different activities. The gamma activity of the

samples was determined using two characteristic ^{22}Na photopeaks at 511 and 1275 keV. The samples were placed in a polyethylene cell (the radioactive face to the bottom) in a well-defined fixed geometry. The activity measurements were carried out for 1000 s. A Genie 2000 program (Canberra Ind., USA) was used for data analysis.

First-Principles Simulations

The DFT calculations of vibrational spectra were carried out using Gaussian 16 software.[s9] The structural optimization and harmonic vibrational frequency calculations were performed for size-limited clusters: CS-$Ga_2S_7H_6$, ES-$Ga_2S_6H_4$, Ga_4S_8H4, CS-$Ga_2Cl_2S_5Na_2H_2$, ES-$Ga_2Cl_2S_4Na_2$, Na_4Cl_4, etc. The Becke three-parameter hybrid exchange functional[s10] and the Lee–Yang–Parr correlation functional (B3LYP)[s11] were applied for these simulations. The small-core relativistic pseudopotential basis set (cc-pVTZ-PP)[s12] and the effective core potentials[s13] were used for cluster geometry optimization and Raman intensity calculations. Most of the structures were optimized using the tight convergence option ensuring adequate convergence and reliability of computed wavenumbers. An extra quadratically convergent self-consistent field procedure[s14] was employed for difficult convergence cases.

Modeling of the diffraction data was carried out using Born–Oppenheimer molecular dynamics implemented within the CP2K package.[s15] The generalized gradient approximation (GGA) and the PBE0 hybrid exchange–correlation functional[s16,s17] combining the exact Hartree–Fock and DFT approaches were used, providing better agreement with experiment. The Grimme dispersion corrections D3BJ[s18] were also employed. The initial atomic configurations for liquid 2NaCl-Ga_2S_3 were created and optimized using the RMC_POT++ code[s19] against the experimental X-ray and neutron data. The size of the cubic simulation box, containing 495 atoms (110 Na, 110 Cl, 110 Ga, and 165 S), was chosen to match the experimental density. Further optimization was carried out using DFT, applying the molecularly optimized correlation consistent polarized triple-zeta valence basis set along with the norm-conserving relativistic Goedecker–Teter–Hutter-type pseudopotentials.[s20] FPMD simulations were performed using a canonical NVT ensemble with a Nosé–Hoover[s21,s22] thermostat. The simulation boxes were heated from 300 K to 1400 K using 100 K or 200 K steps for 30–89 ps each. At 1400 K, the systems were equilibrated for 30 ps and cooled down to 400 K using the 100 K steps for 30–58 ps. The pyMolDyn code[s23] applying the Dirichlet–Voronoi tessellation was used for the calculation of microscopic voids and cavities. Electron density distribution was collected at the same level of theory and stored in Gaussian CUBE file format.[s9] The Bader atomic charges[s24] were calculated using the algorithm for doing analysis on a charge density grid.[s25] The derived charges were averaged over 1 ps for all atoms of the simulation box.

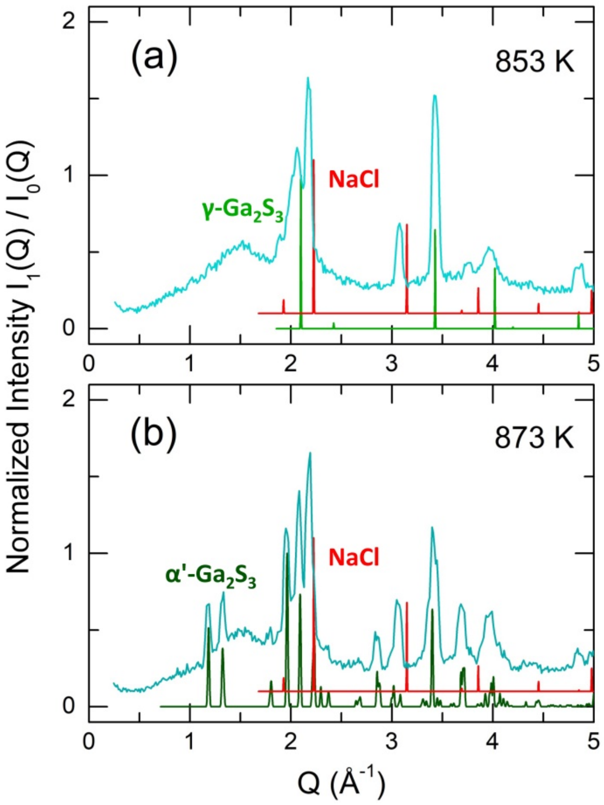

Figure S0. High-energy X-ray diffraction data of 2NaCl-Ga_2S_3 at (a) 853 K and (b) 873 K. The Bragg peaks of crystalline references NaCl, γ-Ga_2S_3 and α'-Ga_2S_3 at room temperature are also shown. The shift of the experimental HE-XRD Bragg peaks to lower Q-values is primarily attributed to thermal expansion, particularly noticeable for NaCl.

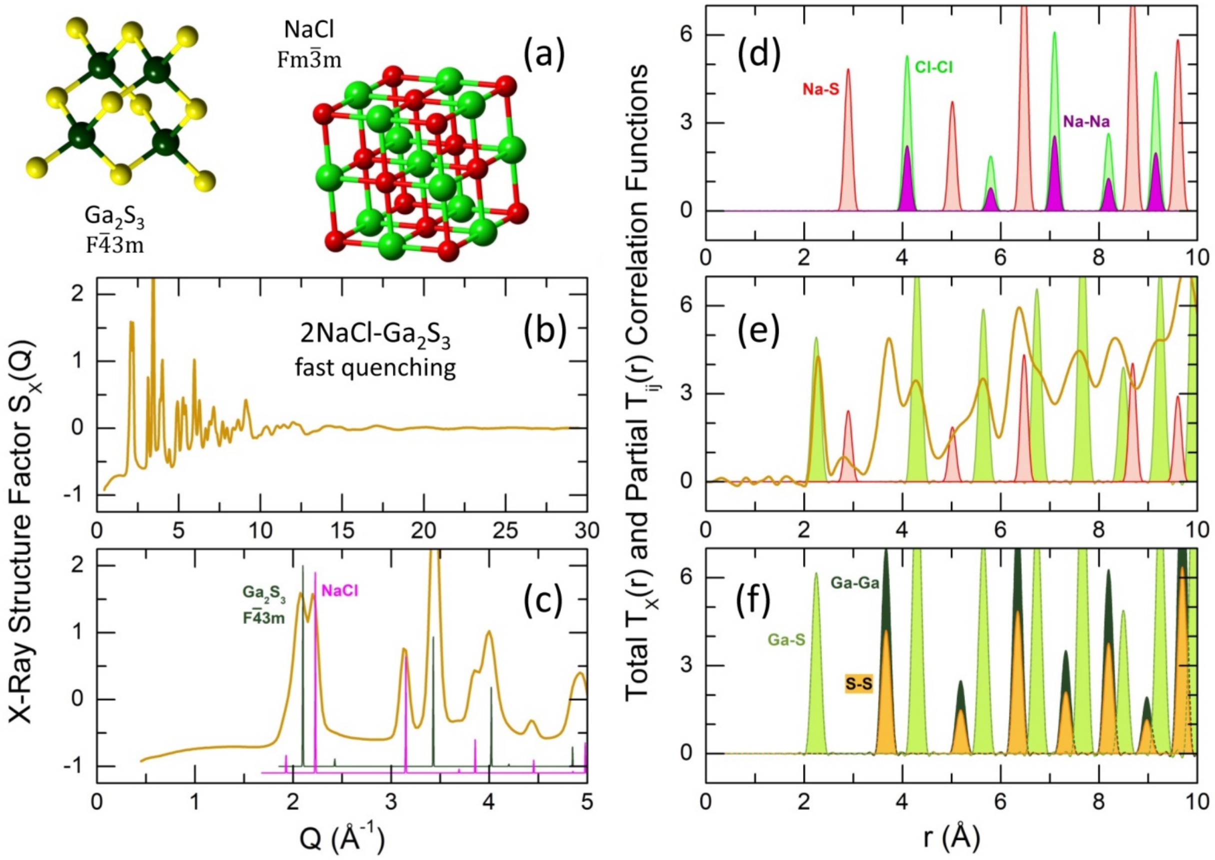

Figure S1. High-energy X-ray diffraction data for rapidly quenched 2NaCl-Ga_2S_3; (a) schematic representation of crystalline lattices for cubic Ga_2S_3 (space group $F\bar{4}3m$) and cubic NaCl (space group $Fm\bar{3}m$); the X-ray structure factor $S_X(Q)$ over (b) an extended Q-range and (c) at low Q < 5 Å^{-1}; the Bragg peaks of cubic Ga_2S_3 and NaCl are also shown in (c); partial $T_{ij}(r)$ and total correlation function $T_X(r)$ in r-space: (d) cubic NaCl; (e) experimental $T_X(r)$ plotted together with $T_{GaS}(r)$ and $T_{NaCl}(r)$ calculated partials for reference crystals; (f) cubic Ga_2S_3. The partial $T_{ij}(r)$ functions were calculated using the XTAL program.[s26]

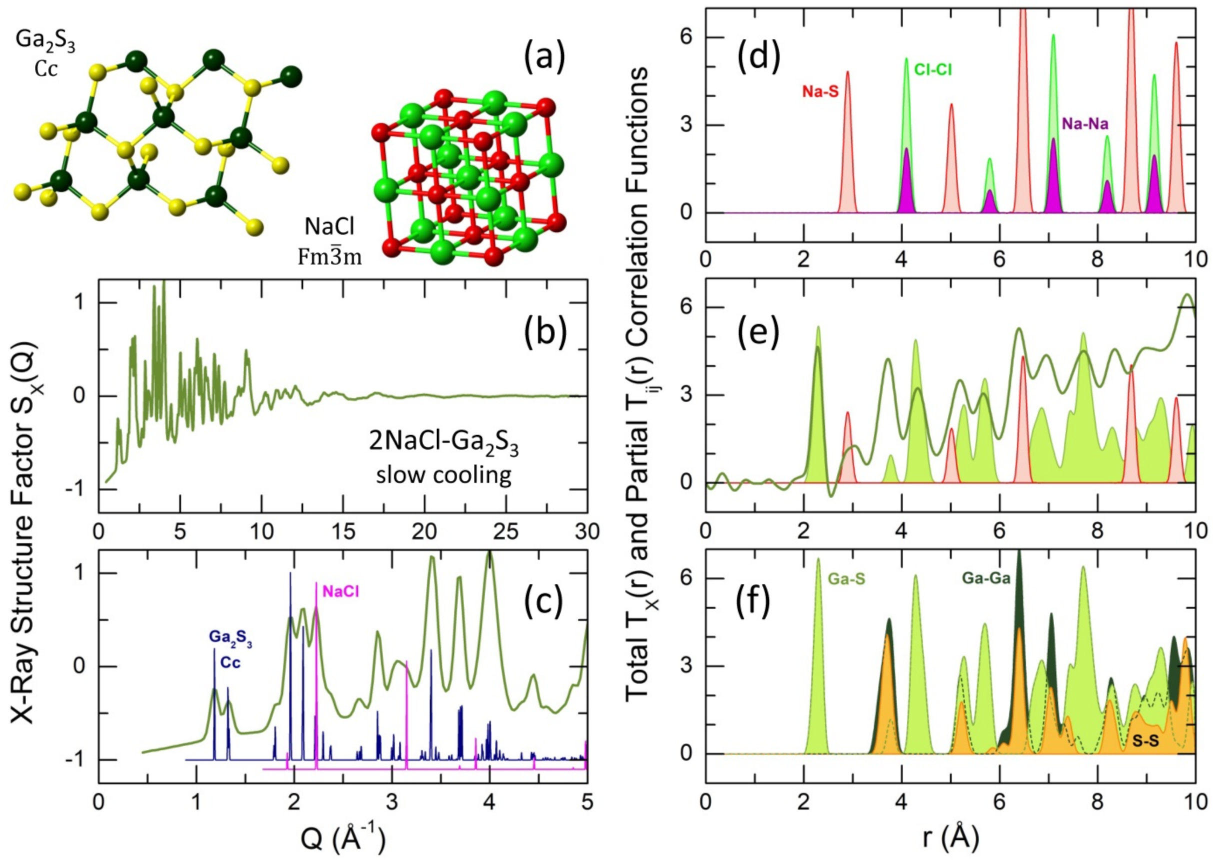

Figure S2. High-energy X-ray diffraction data for slowly cooled 2NaCl-Ga_2S_3; (a) schematic representation of crystalline lattices for monoclinic Ga_2S_3 (space group Cc) and cubic NaCl (space group $Fm\bar{3}m$); the X-ray structure factor $S_X(Q)$ over (b) an extended Q-range and (c) at low Q < 5 Å^{-1}; the Bragg peaks of monoclinic Ga_2S_3 and NaCl are also shown in (c); partial $T_{ij}(r)$ and total correlation function $T_X(r)$ in r-space: (d) cubic NaCl; (e) experimental $T_X(r)$ plotted together with $T_{GaS}(r)$ and $T_{NaCl}(r)$ calculated partials for reference crystals; (f) monoclinic Ga_2S_3. The partial $T_{ij}(r)$ functions were calculated using the XTAL program.[s26]

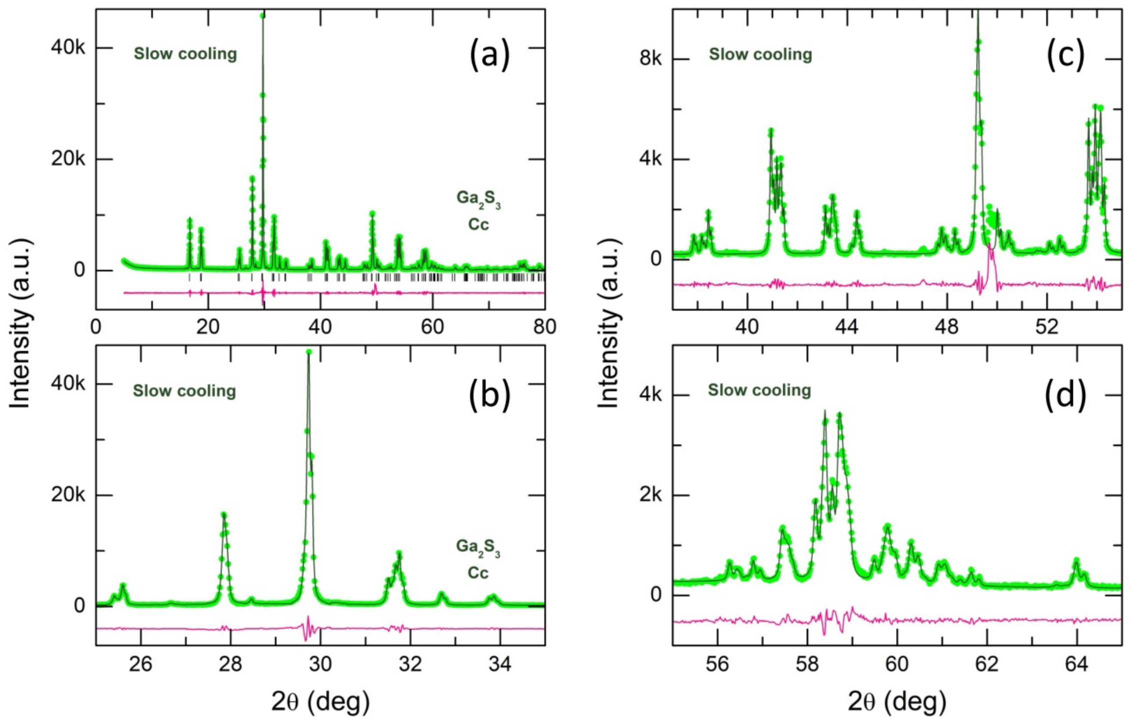

Figure S3. LeBail refinement of slowly cooled 2NaCl-Ga_2S_3 sample following the sodium chloride dissolution in water; (a) the total 2θ-range; refinement details between (b) 25°-35°, (c) 37°-55°, and (d) 55°-65°.

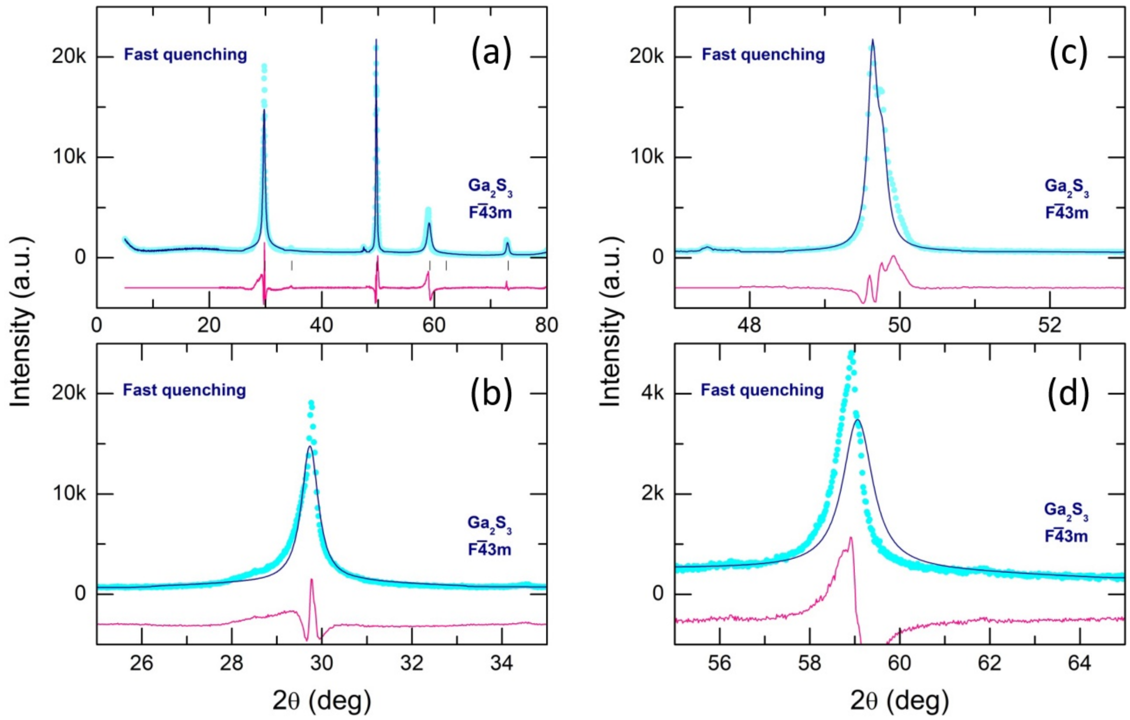

Figure S4. LeBail refinement of fast-quenched 2NaCl-Ga_2S_3 sample, following the sodium chloride dissolution in water and assuming the cubic lattice (space group $F\bar{4}3m$); (a) the total 2θ-range; refinement details between (b) 25°-35°, (c) 47°-53°, and (d) 55°-65°.

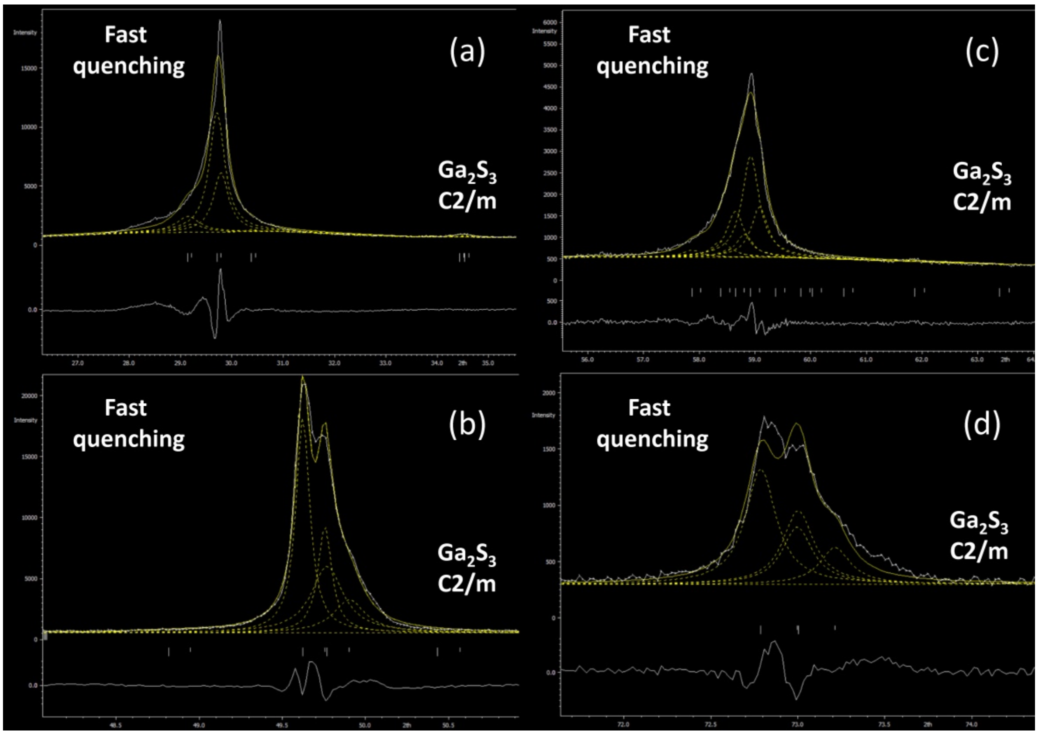

Figure S5. LeBail refinement of fast-quenched 2NaCl-Ga_2S_3 sample, following the sodium chloride dissolution in water and assuming the monoclinic lattice (space group $C2/m$); refinement details between (a) 27°-35°, (b) 48°-51°, (c) 56°-64°, and (d) 72°-74°. This hypothesis yields the best fit with profile agreement factors Rp = 6.88, wRp = 8.72 (GOF = 2.68) for 2782 points and 15 parameters.

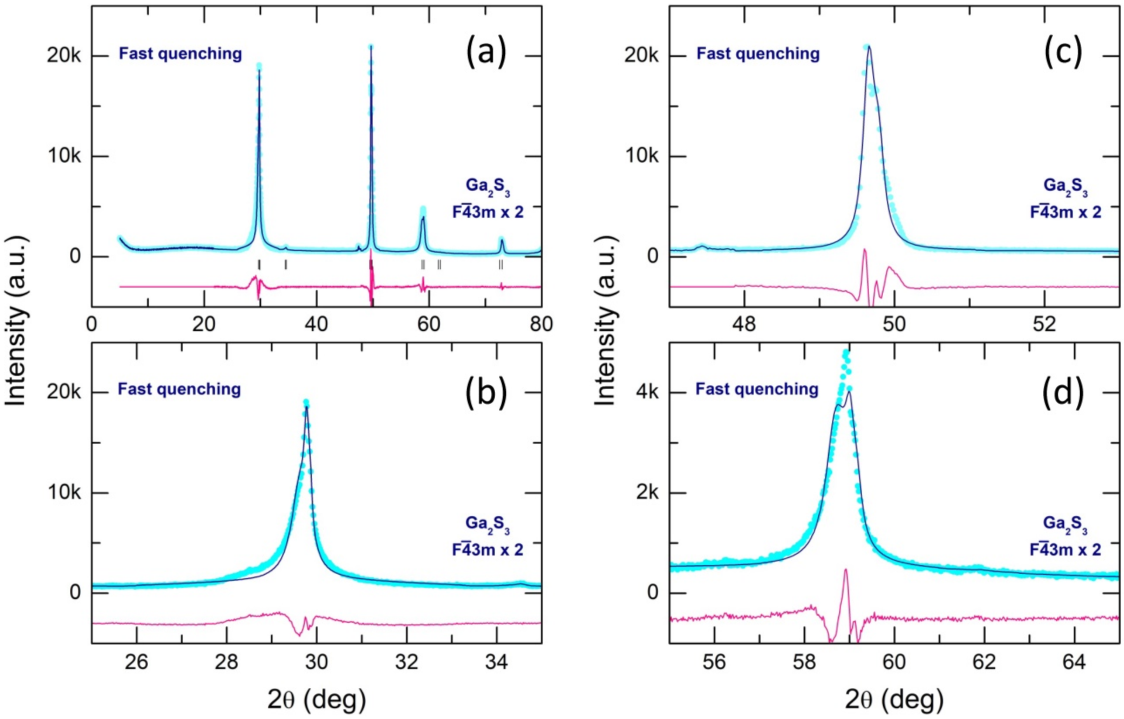

Figure S6. LeBail refinement of fast-quenched 2NaCl-Ga_2S_3 sample, following the sodium chloride dissolution in water and assuming two slightly different cubic polymorphs (space group $F\bar{4}3m$); (a) the total 2θ-range; refinement details between (b) 25°-35°, (c) 47°-53°, and (d) 55°-65°.

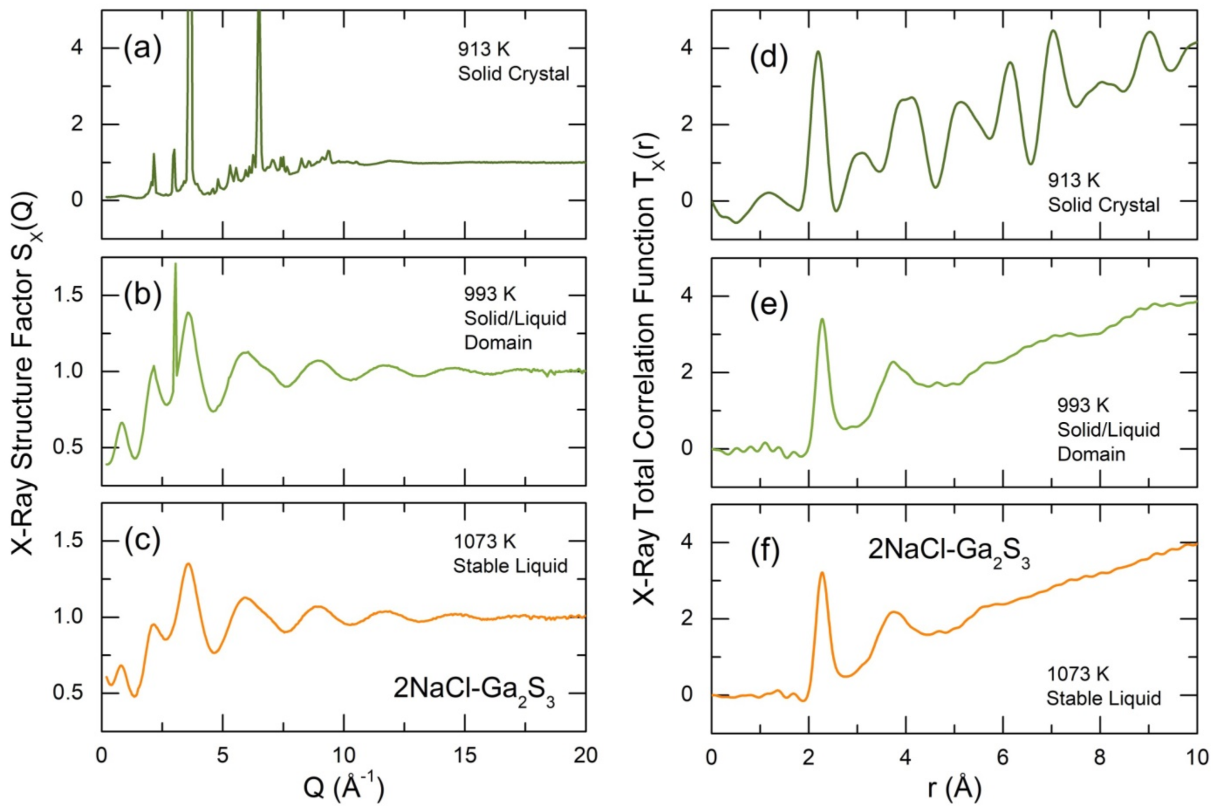

Figure S7. High-energy X-ray diffraction data of 2NaCl-Ga_2S_3 in Q- and r-space in the vicinity of the melting point; the X-ray total structure factor $S_X(Q)$ at (a) 913 K (solid crystal), (b) 993 K (solid/liquid domain above the eutectic temperature), and (c) 1073 K (stable liquid); the X-ray total correlation function $T_X(r)$ at (d) 913 K, (e) 993 K, and (f) 1073 K.

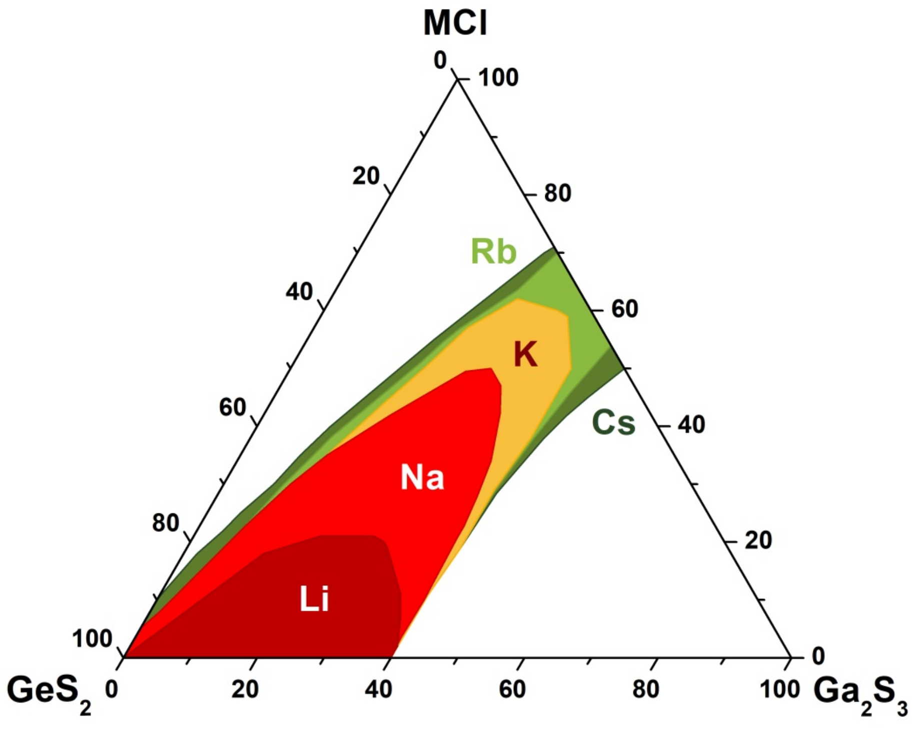

Figure S8. Glass-forming regions in the MCl-Ga_2S_3-GeS_2 systems.[s27,s28]

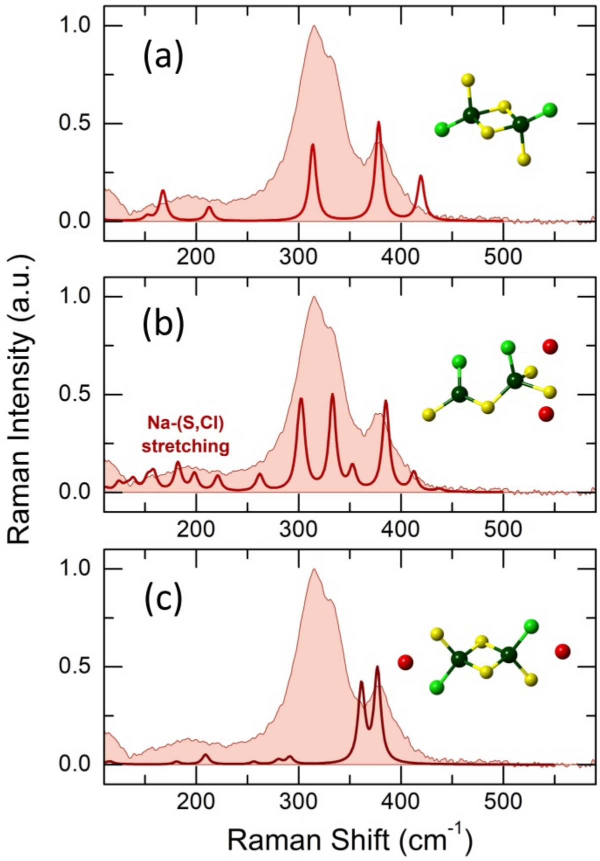

Figure S9. Experimental and DFT Raman spectra of liquid 2NaCl-Ga_2S_3 at 1113 K (highlighted in light red in all panels) and size-limited clusters: (a) ES-$Ga_2Cl_2S_4H_2$, (b) CS-$Ga_2Cl_2S_4Na_2H$, and (c) ES-$Ga_2Cl_2S_4Na_2$. The terminal H species are not shown, and H-related vibrations are removed from the spectra.

Table S1. Assignments of the Most Intense Vibration Frequencies in the DFT Raman Spectra of Size-Limited Clusters.

CS-$Ga_2S_7H_6$

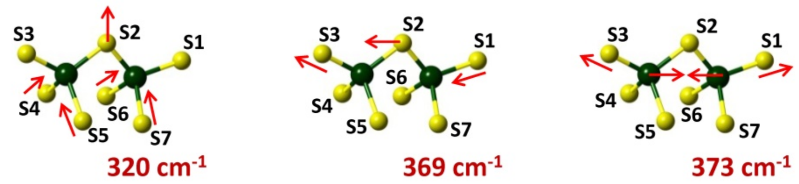

Frequency (cm^{-1})	Assignment
320	Asymmetric in-phase Ga-S stretching, incl. S2, S4, S5, S6, and S7 species
369	Asymmetric in-phase Ga-S stretching, incl. S1, S2, and S3 species
373	Symmetric in-phase Ga-S stretching, incl. S1and S3 species

Table S1. Assignments of the Most Intense Vibration Frequencies in the DFT Raman Spectra of Size-Limited Clusters (continuation).

ES-$Ga_2S_6H_4$

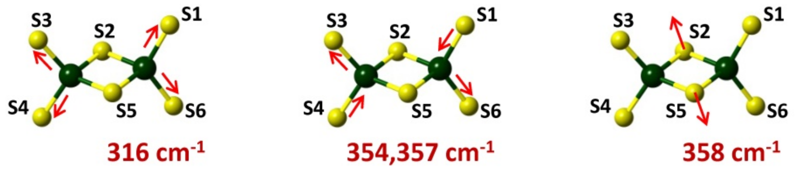

Frequency (cm^{-1})	Assignment
316	Symmetric in-phase Ga-S stretching, incl. S1, S3, S4, and S6 species
354, 357	Asymmetric in-phase Ga-S stretching, incl. either S1 and S6, or S3 and S4 species
358	Breathing of the Ga–S–Ga–S ring, incl. S2 and S5 species

$Ga_4S_8H_4$

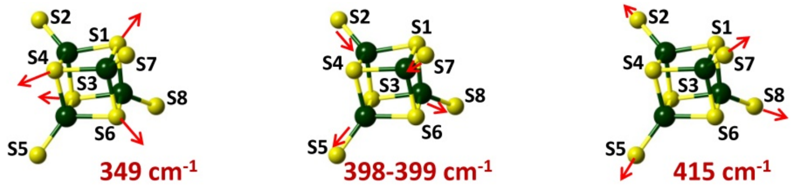

Frequency (cm^{-1})	Assignment
349	Breathing of the Ga_4S_4 cubic entity, incl. S1, S3, S4, and S6 species
398-399	Asymmetric in-phase Ga-S stretching, incl. S2, S5, S7, and S8 species species
415	Symmetric in-phase Ga-S stretching, incl. S2, S5, S7, and S8 species species

CS-$Ga_2Cl_2S_5Na_2H_2$

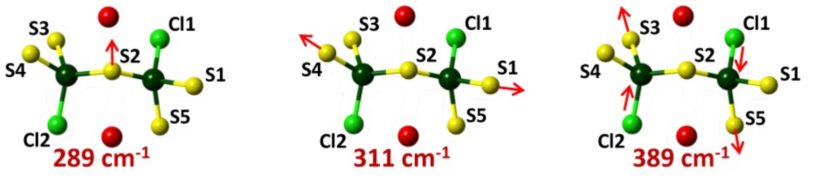

Frequency (cm^{-1})	Assignment
289	Symmetric Ga-S stretching, incl. S2 species
311	Symmetric in-phase Ga-S stretching, incl. S1 and S4 species
389	Symmetric in-phase Ga-S and Ga-Cl vibrations, incl. S3, S5, Cl1, and Cl2 species, which are asymmetric vis-à-vis each other

Na_4Cl_4

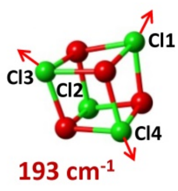

Frequency (cm^{-1})	Assignment
193	Breathing of the Na4Cl4 cage, including all Na and Cl species

^{22}Na Tracer Diffusion Profiles

Penetration profiles for ^{22}Na tracer diffusion in the NaCl-Ga_2S_3-GeS_2 glasses obey the usual solution of Fick's law for an infinitesimally thin deposit of radioactive isotope on a semi infinite specimen[s29]

$$1 - \frac{A(y,t)}{A_0} = \mathrm{erf}(q)\,, \tag{s1}$$

where

$$q = \frac{y}{2\sqrt{D_{\mathrm{Na}} t}}\,, \tag{s2}$$

$A(y,t)$ is the residual radioactivity of the sample after a thickness y was removed, t is the diffusion anneal time, A_0 is the initial residual activity, D_{Na} is the sodium tracer diffusion coefficient, and $\mathrm{erf}(q)$ is the Gauss error function. Experimentally determined values of $A(y,t)$ and A_0 yield q values which, when plotted vs. y, produce a straight line passing through the origin (Figure S10).

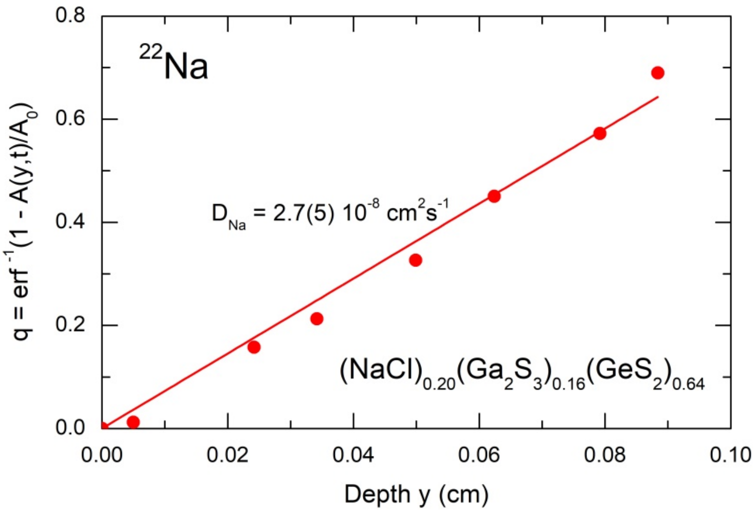

Figure S10. ^{22}Na tracer diffusion profile in a $(NaCl)_{0.20}(Ga_2S_3)_{0.16}(GeS_2)_{0.64}$ glass after annealing at 492 K.

The determined sodium diffusion coefficients D_{Na} obey the Arrhenius relation (Figure 10a), allowing to extract the diffusion parameters: the activation energy E_{d}, pre-exponential factor D_0 and diffusivity at 298 K

$$D_{\mathrm{Na}} = D_0 \exp\left(-\frac{E_{\mathrm{d}}}{kT}\right). \tag{s3}$$

The derived diffusion and conductivity parameters were applied for extrapolation to the 2NaCl-Ga_2S_3 composition (Figure S11).

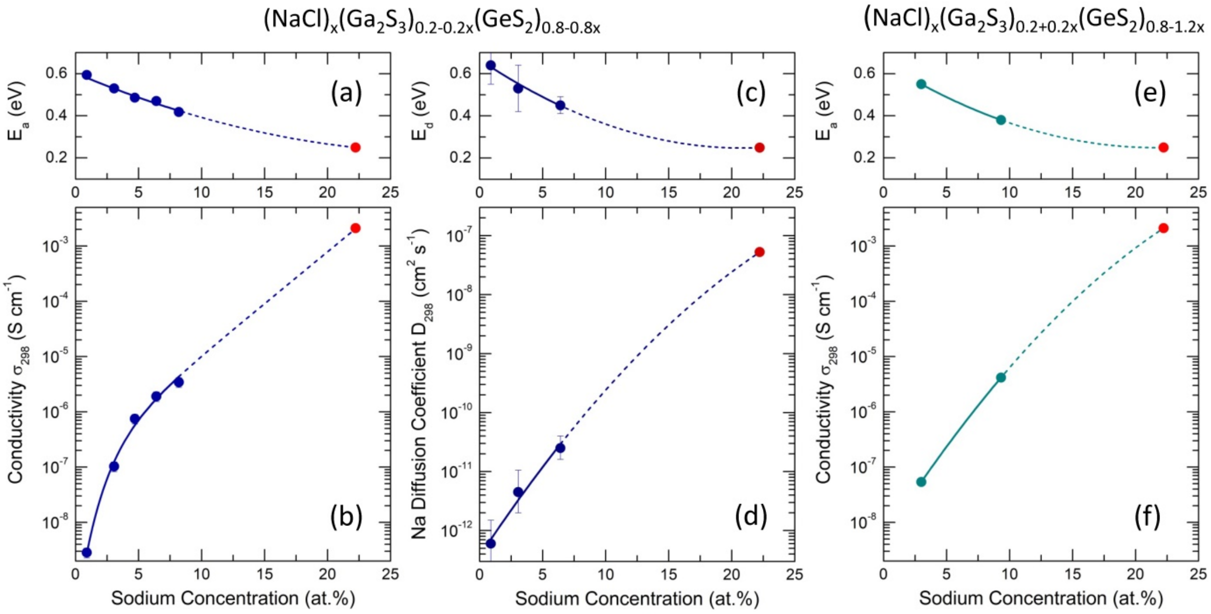

Figure S11. Conductivity and [22]Na tracer diffusion parameters plotted as a function of sodium concentration in the NaCl-Ga_2S_3-GeS_2 system: (a) conductivity activation energy E_a and (b) room-temperature conductivity σ_{298} for the $(\mathrm{NaCl})_x(\mathrm{Ga_2S_3})_{0.2-0.2x}(\mathrm{GeS_2})_{0.8-0.8x}$ tie line; (c) diffuson activation energy E_d and (d) room-temperature diffusivity D_{298} for the $(\mathrm{NaCl})_x(\mathrm{Ga_2S_3})_{0.2-0.2x}(\mathrm{GeS_2})_{0.8-0.8x}$ tie line; (e) conductivity activation energy E_a and (f) room-temperature conductivity σ_{298} for the $(\mathrm{NaCl})_x(\mathrm{Ga_2S_3})_{0.2+0.2x}(\mathrm{GeS_2})_{0.8-1.2x}$ tie line. The red circle corresponds to the sodium concentration in the hypothetical glassy 2NaCl-Ga_2S_3.

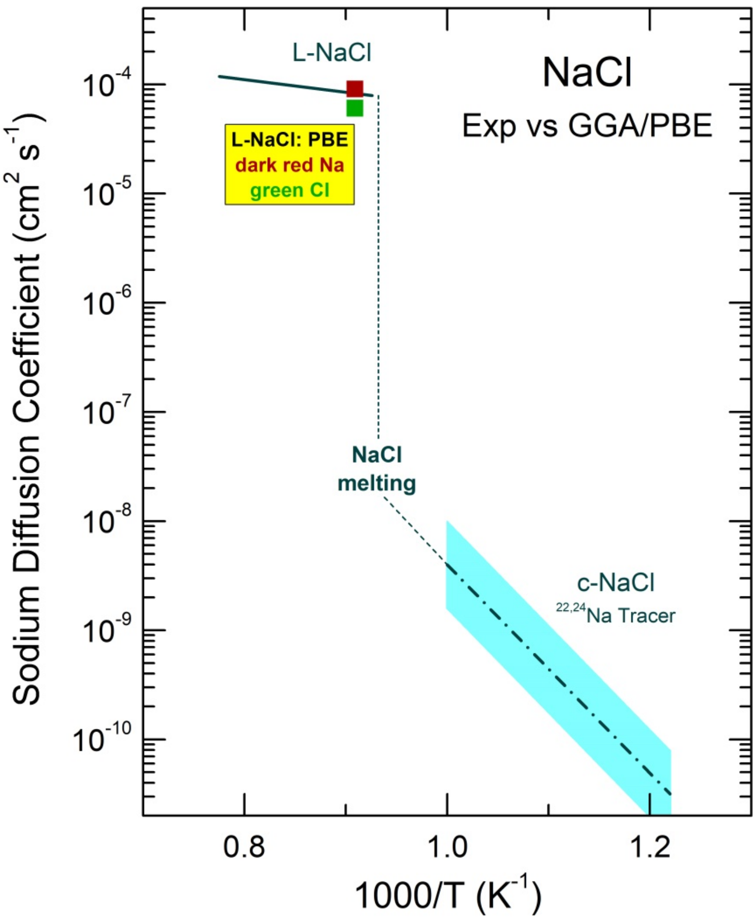

Figure S12. Experimental[s30-s35] and FPMD-derived (our preliminary data) sodium diffusion coefficients D_{Na} in solid and liquid NaCl. The D_{Na} in liquid sodium chloride was recalculated from the ionic conductivity.[s30]

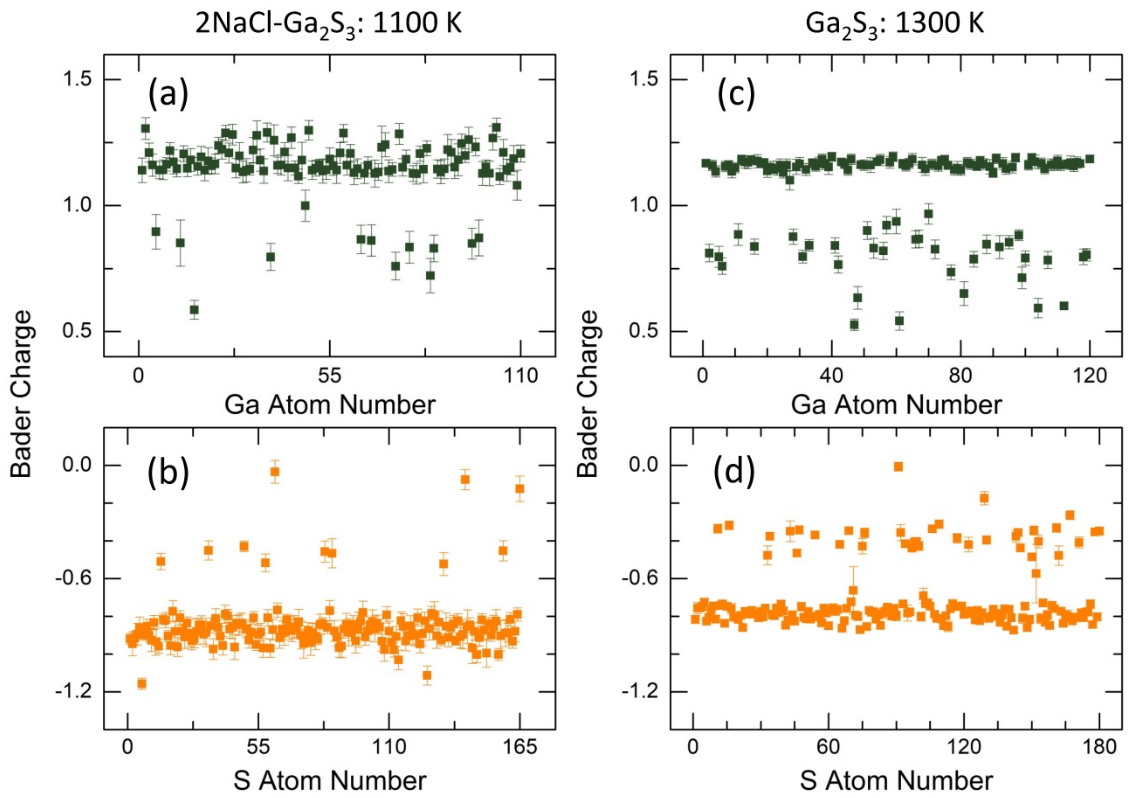

Figure S13. FPMD-derived Bader charges q_{Ga} and q_{S} in (a,b) liquid 2NaCl-Ga_2S_3 at 1100 K, and (c,d) liquid Ga_2S_3 at 1300 K (our preliminary results).

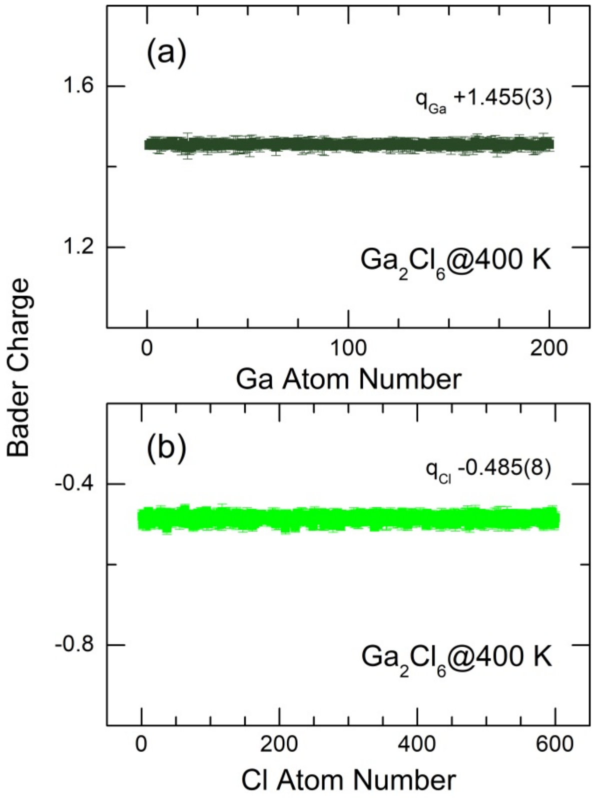

Figure S14. FPMD-derived Bader charges (a) q_{Ga} and (b) q_{Cl} in liquid gallium trichloride at 400 K (our preliminary results).

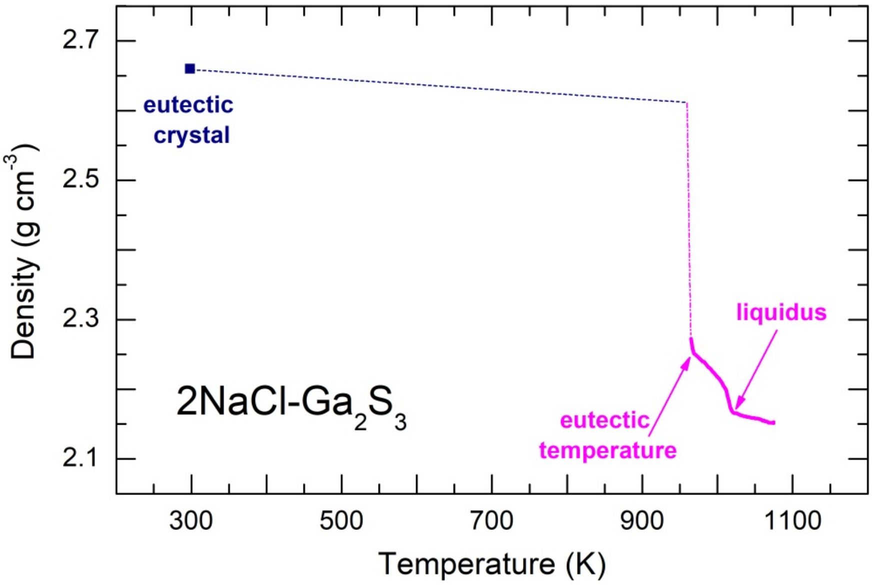

Figure S15. Experimental density change upon solidification of liquid 2NaCl-Ga_2S_3, measured using X-ray transmission experiment.

Additional References

[s1] A. C. Hannon, *Nucl. Instrum. Methods A* **2005**, *551*, 88.

[s2] A. C. Hannon, W. S. Howells, A. K. Soper, *Inst. Phys. Conf. Ser*. **1990**, *107*, 193.

[s3] K. Ohara, Y. Onodera, M. Murakami, S. Kohara, *J. Phys.: Condens. Matter* **2021**, *33,* 383001.

[s4] S. Kohara, M. Itou, K. Suzuya, Y. Inamura, Y. Sakurai, Y. Ohishi, M. Takata, *J. Phys.: Condens. Matter* **2007**, *19*, 506101.

[s5] A. P. Hammersley, S. O. Svensson, M. Hanfland, A. N. Fitch, D. Häusermann, *High Press. Res*. **1996**, *14*, 235.

[s6] L. B. Skinner, C. J. Benmore, J. B. Parise, *Nucl. Instrum. Methods Phys. Res*. **2012**, *662*, 61.

[s7] S. Gates-Rector, T. Blanton, *Powder Diffr*. **2019**, *34*, 352.

[s8] V. Petricek, M. Dusek, L. Palatinus, *Z. Kristallogr*. **2014**, *229*, 345.

[s9] M. J. Frisch, G. W. Trucks, H. B. Schlegel, G. E. Scuseria, M. A. Robb, J. R. Cheeseman, G. Scalmani, V. Barone, G. A. Petersson, H. Nakatsuji, X. Li, M. Caricato, A. V. Marenich, J. Bloino, B. G. Janesko, R. Gomperts, B. Mennucci, H. P. Hratchian, J. V. Ortiz, A. F. Izmaylov, J. L. Sonnenberg, D. Williams-Young, F. Ding, F. Lipparini, F. Egidi, J. Goings, B. Peng, A. Petrone, T. Henderson, D. Ranasinghe, V. G. Zakrzewski, J. Gao, N. Rega, G. Zheng, W. Liang, M. Hada, M. Ehara, K. Toyota, R. Fukuda, J. Hasegawa, M. Ishida, T. Nakajima, Y. Honda, O. Kitao, H. Nakai, T. Vreven, K. Throssell, J. A. Montgomery, Jr., J. E. Peralta, F. Ogliaro, M. J. Bearpark, J. J. Heyd, E. N. Brothers, K. N. Kudin, V. N. Staroverov, T. A. Keith, R. Kobayashi, J. Normand, K. Raghavachari, A. P. Rendell, J. C. Burant, S. S. Iyengar, J. Tomasi, M. Cossi, J. M. Millam, M. Klene, C. Adamo, R. Cammi, J. W. Ochterski, R. L. Martin, K. Morokuma, O. Farkas, J. B. Foresman, D. J. Fox, *Gaussian 16, revision B.01*; Gaussian, Inc.: Wallingford, CT, 2016.

[s10] A. D. Becke, *J. Chem. Phys*. **1993**, *98*, 5648.

[s11] C. Lee, W. Yang, R. G. Parr, *Phys. Rev. B* **1988**, *37*, 785.

[s12] D. Feller, *J. Comput. Chem*. **1996**, *17*, 1571.

[s13] K. A. Peterson, D. Figgen, E. Goll, H. Stoll, M. Dolg, *J. Chem. Phys*. **2003**, *119*, 11113.

[s14] G. B. Bacskay, *Chem. Phys*. **1981**, *61*, 385.

[s15] T. D. Kühne, M. Iannuzzi, M. Del Ben, V. V. Rybkin, P. Seewald, F. Stein, T. Laino, R. Z. Khaliullin, O. Schütt, F. Schiffmann, D. Golze, J. Wilhelm, S. Chulkov, M. H. Bani-Hashemian, V. Weber, U. Borštnik, M. Taillefumier, A. S. Jakobovits, A. Lazzaro, H. Pabst, T. Müller, R. Schade, M. Guidon, S. Andermatt, N. Holmberg, G. K. Schenter, A. Hehn, A. Bussy, F. Belleflamme, G. Tabacchi, A. Glöß, M. Lass, I. Bethune, C. J. Mundy, C. Plessl, M. Watkins, J. VandeVondele, M. Krack, J. Hutter, *J. Chem. Phys*. **2020**, *152*, 194103.

[s16] J. P. Perdew, M. Ernzerhof, K. Burke, *J. Chem. Phys*. **1996**, *105*, 9982.

[s17] C. Adamo, V. Barone, *J. Chem. Phys*. **1999**, *110*, 6158.

[s18] S. Grimme, S. Ehrlich, L. Goerigk, *J. Comput. Chem*. **2011**, *32*, 1456.

[s19] O. Gereben, L. Pusztai, *J. Comput. Chem*. **2012**, *33*, 2285.

[s20] C. Hartwigsen, S. Goedecker, J. Hutter, *Phys. Rev. B* **1998**, *58*, 3641.

[s21] S. Nosé, *Mol. Phys*. **1984**, *52*, 255.

[s22] W. G. Hoover, *Phys. Rev. A* **1985**, *31*, 1695.

[s23] I. Heimbach, F. Rhiem, F. Beule, D. Knodt, J. Heinen, R. O. Jones, *J. Comput. Chem*. **2017**, *38*, 389.

[s24] R. F. W. Bader, *Atoms in Molecules: A Quantum Theory*, Clarendon Press, Oxford, 1994.

[s25] G. Henkelman, A. Arnaldsson, H. Jónsson, *Comput. Mater. Sci*. **2006**, *36*, 354.

[s26] A. C. Hannon, *XTAL: a Program for Calculating Interatomic Distances and Coordination Numbers for Model Structures*, Rutherford-Appleton Laboratory Report RAL-93-063; 1993, http://wwwisis2.isis.rl.ac.uk/disordered/ACH/Software/xtal.htm.

[s27] Z. U. Borisova, E. A. Bychkov, Y. S. Tveryanovich, *Interaction of Metals with Chalcogenide Glasses*, Leningrad University Press, Leningrad, 1991; pp. 88–102.

[s28] A. Tverjanovich, Y. S. Tveryanovich, S. Loheider, *J. Non-Cryst. Solids* **1996**, *208*, 49.

[s29] J. Crank, *The Mathematics of Diffusion,* 2nd ed., Clarendon Press, Oxford, 1975, pp. 28–43.

[s30] G. J. Janz, R. P. T. Tomkins, C. B. Allen, J. R. Downey Jr., G. L. Garner, U. Krebs, S. K. Singer, *J. Phys. Chem. Reference Data* **1975**, *4*, 871.

[s31] D. Mapother, H. N. Crooks, R. Maurer, *J. Chem. Phys*. **1950**, *18*, 1231.
[s32] V. C. Nelson, R. J. Friauf, *J. Phys. Chem. Solids* **1970**, *31*, 825.
[s33] H. L. Downing Jr., R. J. Friauf, *J. Phys. Chem. Solids* **1970**, *31*, 845.
[s34] F. Bénière, M. Bénière, M. Chemla, *J. Phys. Chem. Solids* **1970**, *31*, 1205.
[s35] S. J. Rothman, N. L. Peterson, A. L. Laskar, L. C. Robinson, *J. Phys. Chem. Solids* **1972**, *33*, 1061.
